# Targeted next-generation sequencing detects novel gene–phenotype associations and expands the mutational spectrum in cardiomyopathies

**DOI:** 10.1371/journal.pone.0181842

**Published:** 2017-07-27

**Authors:** Cinzia Forleo, Anna Maria D’Erchia, Sandro Sorrentino, Caterina Manzari, Matteo Chiara, Massimo Iacoviello, Andrea Igoren Guaricci, Delia De Santis, Rita Leonarda Musci, Antonino La Spada, Vito Marangelli, Graziano Pesole, Stefano Favale

**Affiliations:** 1 Cardiology Unit, Department of Emergency and Organ Transplantation, University of Bari Aldo Moro, Bari, Italy; 2 Department of Biosciences, Biotechnology and Biopharmaceutics, University of Bari Aldo Moro, Bari, Italy; 3 Institute of Biomembranes, Bioenergetics and Molecular Biotechnologies (IBIOM), National Research Council, Bari, Italy; 4 Department of Biosciences, University of Milano, Milano, Italy; Indiana University, UNITED STATES

## Abstract

Cardiomyopathies are a heterogeneous group of primary diseases of the myocardium, including hypertrophic cardiomyopathy (HCM), dilated cardiomyopathy (DCM), and arrhythmogenic right ventricular cardiomyopathy (ARVC), with higher morbidity and mortality. These diseases are genetically diverse and associated with rare mutations in a large number of genes, many of which overlap among the phenotypes. To better investigate the genetic overlap between these three phenotypes and to identify new genotype–phenotype correlations, we designed a custom gene panel consisting of 115 genes known to be associated with cardiomyopathic phenotypes and channelopathies. A cohort of 38 unrelated patients, 16 affected by DCM, 14 by HCM and 8 by ARVC, was recruited for the study on the basis of more severe phenotypes and family history of cardiomyopathy and/or sudden death. We detected a total of 142 rare variants in 40 genes, and all patients were found to be carriers of at least one rare variant. Twenty-eight of the 142 rare variants were also predicted as potentially pathogenic variants and found in 26 patients. In 23 out of 38 patients, we found at least one novel potential gene–phenotype association. In particular, we detected three variants in *OBSCN* gene in ARVC patients, four variants in *ANK2* gene and two variants in *DLG1*, *TRPM4*, and *AKAP9* genes in DCM patients, two variants in *PSEN2* gene and four variants in *AKAP9* gene in HCM patients. Overall, our results confirmed that cardiomyopathic patients could carry multiple rare gene variants; in addition, our investigation of the genetic overlap among cardiomyopathies revealed new gene–phenotype associations. Furthermore, as our study confirms, data obtained using targeted next-generation sequencing could provide a remarkable contribution to the molecular diagnosis of cardiomyopathies, early identification of patients at risk for arrhythmia development, and better clinical management of cardiomyopathic patients.

## Introduction

Inherited cardiomyopathies are a group of heart muscle diseases characterized by heterogeneous phenotypes, including dilated cardiomyopathy (DCM), hypertrophic cardiomyopathy (HCM), and arrhythmogenic right ventricular cardiomyopathy (ARVC), with higher morbidity and mortality [1, 2]. DCM, defined by the presence of left ventricular (LV) or biventricular dilatation and systolic dysfunction in absence of hypertension, valvular disease, or coronary artery disease [1, 2], is one of the leading causes of heart failure (HF) and sudden death (SD) and represents the most common condition requiring a heart transplant [1, 2]. HCM, characterized by increased LV wall thickness that is not solely explained by abnormal loading conditions [3, 4], is the most common cause of SD in young, and is considered an important cause of HF and embolic stroke secondary to atrial fibrillation [5, 6]. A diagnosis of ARVC is made mainly based on functional and structural abnormalities of the right ventricle (RV), fibro-fatty replacement of the myocardium, depolarization and repolarization alterations, arrhythmias with left bundle branch block (LBBB) morphology, and family history, with no single gold standard exam of diagnosis established to date [7]. ARVC represents one of the major causes of SD in the young and athletes [8, 9]; rarely, RV or biventricular dysfunction leads to HF [10]. Phenotypic overlap between different cardiomyopathies has been well described, leading to diagnostic uncertainty in some cases [11].

Over the past 20 years, substantial progress in the understanding of the genetic basis of cardiomyopathies has been made [1–3, 12, 13]. Recent family screening studies showed that autosomal dominant inheritance was the predominant pattern of transmission up to 48% of DCM cases; possibilities of X-linked, autosomal-recessive, and mitochondrial inheritance were less common [14]. To date, genes that encode sarcomere, cytoskeleton, desmosomes, nuclear envelop proteins, and ion channels have been found to be associated with DCM [15, 16], with TTN being the most frequently associated gene [17]. Current studies show that up to 60% of HCM patients exhibit an autosomal-dominant trait with mutations in genes mostly affecting sarcomere proteins [3, 18–20]. Moreover, mutations in genes that encode Z-disc proteins or intracellular calcium modulators have also been detected [3]. The classical inheritance pattern of ARVC is autosomal-dominant with variable expression and age-related penetrance [21]. To date, most of genes that have been found associated with the ARVC phenotype encode desmosomal proteins and cause disease in up to 50% of probands [12, 21]. However, non-desmosomal genes have also been implicated in ARVC development [12]. In addition to the genetic heterogeneity, a wide genetic overlap has also been noted among different cardiomyopathic phenotypes, and between these heart muscle disorders and channelopathies [22–29].

According to recent international guidelines, genetic testing is strongly recommended for each patient with inherited cardiomyopathy to identify a causative mutation and subsequently provide pre-symptomatic testing of relatives who are at risk of developing the same disease at a later stage; moreover, some disease-causing gene mutations have been found to be associated with more severe clinical features, presentation at an early age, overall poor prognosis, or increased risk of SD [30–34]. Therefore, genetic testing can have considerable implications in terms of early diagnosis, implementation of prognostic stratification algorithms, and timely therapeutic interventions [32, 35].

Sanger sequencing is a standard technique of molecular diagnostics for disorders predominantly related to a single causative gene. However, this screening method is laborious. The high degree of genetic heterogeneity, with more than 100 cardiomyopathy-related genes identified to date, can be addressed more efficiently by high-throughput sequencing, referred to as next-generation sequencing (NGS) [36], which includes targeted resequencing, whole exome sequencing, and whole genome sequencing. Targeted enrichment appears to be the method of choice in a clinical diagnostic setting, not only because it allows focusing on the genes relevant to a particular disorder, thereby preventing unsolicited findings, but also because it provides a superior quality of exon representation and coverage.

In this study, we developed a custom “pan-cardiomyopathy panel” containing 115 genes known to be associated with DCM, HCM, and ARVC as well as to channelopathies. Based on this panel, we performed molecular screening in 38 unrelated patients, 16 affected by DCM, 14 by HCM, and 8 by ARVC, to better investigate the genetic overlap between these three phenotypes and to identify new gene–phenotype associations using the Illumina MiSeq platform. Moreover, we investigated the possible role of genes linked to channelopathies in modifying the clinical cardiomyopathic phenotype.

The results of this study put a spotlight on novel cardiomyopathic gene–phenotype associations, extending the mutational spectrum underlying cardiomyopathies. Furthermore, our data suggest that some genes involved in channelopathies may act as genetic modifiers, modulating the clinical features and severity of cardiomyopathic phenotypes, as well as contributing to the variable cardiomyopathic phenotypic expression. Our findings support the “pan-cardiomyopathy panel” approach, since many patients carried multiple rare variants in several genes.

## Materials and methods

### Ethics statement

All patients provided their written informed consent to participate in this study. We obtained written consent from parents of the minors included in the study. The project conformed to the principles of the Declaration of Helsinki (World Medical Association) and was approved by the Ethics Committee of the University Hospital Consortium, Policlinico of Bari, Italy.

### Study subjects, disease criteria, and clinical evaluation

The patients who were referred to the Cardiomyopathy Unit, Cardiology Unit, Department of Emergency and Organ Transplantation, University of Bari Aldo Moro, Bari (Italy), between February 2008 and June 2014 were enrolled in this study. A total of 38 Italian unrelated patients (16 DCM, 14 HCM, and 8 ARVC) were recruited on the basis of more severe phenotypes and family history of cardiomyopathy and/or SD (S1 Table).

All patients underwent clinical work-up, including medical history, physical examination, 12-lead electrocardiogram (ECG), transthoracic echocardiography, and 24-hour ECG monitoring. Where appropriate, exercise testing and coronary angiography were performed. Subjects without contraindications, such as pacemaker, defibrillator, or severe claustrophobia, underwent cardiac magnetic resonance imaging.

The diagnosis of DCM was according to the World Health Organization criteria [37] and international guidelines [1, 38]. The exclusion criteria were previously described [39–41]. The diagnosis of HCM was based on international criteria [42, 43]. The diagnosis of ARVC was according to the original [44] and modified [45] Task Force criteria.

### Gene panel design

The gene panel was designed using the Design Studio Tool (Illumina, San Diego, CA, USA). The coding regions and intron–exon boundaries of 115 genes, known to be associated with DCM, HCM, and ARVC as well as channelopathies, were selected for targeted gene enrichment. For genes with multiple transcripts, all exons included in transcripts expressed in cardiac muscle were considered in the gene panel design. The coordinates of genomic regions were based on NCBI build 37 (UCSC hg19). The complete list of genes and transcripts is reported in S2 Table.

### Targeted gene enrichment and high-throughput sequencing

Total DNA was extracted from peripheral blood samples using the Wizard Genomic DNA Purification Kit (Promega, Mannheim, Germany) according to the manufacturer’s instructions, quantified, and qualitatively checked using NanoDrop 2000c (Thermo Fisher Scientific, Waltham, MA, USA).

Custom targeted gene enrichment and DNA library preparation were performed using the Nextera Capture Custom Enrichment kit (Illumina) according to the manufacturer’s instructions. The targeted regions were sequenced using the Illumina MiSeq platform, generating approximately two millions of 150-bp paired-end reads for each sample (Q30 ≥90%).

Sequencing and genotyping data were submitted to “The European Genome-phenome Archive” EGA (http://www.nature.com/ng/journal/v47/n7/full/ng.3312.html), with the accession number EGAS00001002506. S3 Table reports the accession numbers of the sequencing data for each patient.

### Variant calling, filtering, and classification

Variant calling was performed using the web tool Online Deep Exome Sequencing Software Analysis (ODESSA) [46]. The pipeline, after sequencing data submission, executed the following steps: the quality checks and filter of the reads; the alignment on the reference genome hg19; the variant preprocessing; the coverage statistics and metrics; the variant calling; the variant annotation. Genetic variants predicted to alter the protein, such as non-synonymous variants, nonsense variants, canonical splicing site variants (affecting the donor or acceptor splice sites), in-frame and frameshift insertion/deletions, supported by at least 10 reads, and with a minor allele frequency (MAF) ≤ 0,4%, ≤ 0,2%, ≤ 0,05% for DCM, HCM and ARVC respectively in variant databases, chosen considering the prevalence of the phenotypes in the general population [47], were selected. To assess the potential functional impacts of variants, four bioinformatics algorithms were used: PolyPhen-2 (PP2), Sorting Tolerant From Intolerant (SIFT), Protein variation effect analyzer (Provean), and Mutation Taster. PhyloP and PhastCons scores were also used to analyze the degree of sequence conservation of the mutated loci, and Grantham’s score was considered to categorize codon replacements. To calculate these three parameters for each rare missense variant, we employed Mutation Taster. Missense variants were considered “potentially pathogenic” if classified simultaneously as “damaging” by SIFT, “deleterious” by Provean, “possibly” or “probably damaging” by Polyphen-2, and “Disease Causing” by Mutation Taster. *TTN* missense variants were not considered potentially pathogenic independently from the predictors’ results because, as recently reported, there is no statistical difference in frequency between cases and controls and their potentially pathogenic role can be established only by a segregation analysis in affected families [48, 49]. Stopgain and stoploss variants, splicing variants, frameshift, and in-frame insertions and deletions were considered potentially pathogenic if classified as “Disease Causing” by Mutation Taster; we defined these variant types as Radical variants. *TTN* truncating variants were considered potentially pathogenic only if they affected TTN isoforms in the A-band region [49–51].

Variants not classified as potentially pathogenic by predictors, but for which a pathogenic role was supported by published data [52] and/or by evidences in ClinVar and HGMD databases were considered as potentially pathogenic. Variants classified as Variants of Uncertain Significance (“VUS”), “Likely Benign” or “Benign” in ClinVar database were not considered as potentially pathogenic variants (S1 Fig).

It should be considered that in our study the designation of potentially pathogenic variants derived from a genetic test aimed to assess new gene-phenotype associations. The clinical relevance of these potentially pathogenic variants should instead be assessed on the basis of the current guidelines [53], but it was not the aim of the study.

### Variant databases and prediction programs

*Variant databases*: 1000 Genomes Project (http://browser.1000genomes.org/index.html), NCBI dbSNP (http://www.ncbi.nlm.nih.gov/SNP/), NHLBI Exome Sequencing Project (ESP) Exome Variant Server (http://evs.gs.washington.edu/EVS/), Genome Aggregation Database (gnomAD; http://gnomad.broadinstitute.org), ClinVar (http://www.ncbi.nlm.nih.gov/clinvar/), Human Genome Mutation Database (HGMD Professional 2014; http://www.biobase-international.com/).

*Prediction programs*: PolyPhen-2 (http://genetics.bwh.harvard.edu/pph2/), SIFT and Provean (http://provean.jcvi.org/protein_batch_submit.php?species=human), and Mutation Taster (http://www.mutationtaster.org/).

## Results

### Clinical features

The clinical characteristics of all evaluated patients are shown in S1 Table.

A total of 16 DCM patients (14 men, 2 women; mean age 38 ± 15 years; range: 15 to 67 years) were included in this study, 11 of which had a family history of DCM and/or SD.

Fourteen patients (7 men and 7 women; mean age 35 ± 14 years; range: 16 to 58 years) presented with either obstructive (n = 7) or non-obstructive (n = 7) form of HCM, 10 of which had a family history of the condition and/or SD.

Eight patients (5 men and 3 women; mean age 40 ± 21 years; range: 12 to 72 years) presented with ARVC, 7 of which had a family history of arrhythmogenic cardiomyopathy and/or SD.

Overall, 31 patients out of 38 received an implantable cardioverter defibrillator (ICD), 24 of which in primary prevention and 7 in secondary prevention; one DCM patient aged 45 years underwent a heart transplant; and one DCM patient aged 71 years carrying an ICD died due to refractory HF.

### Pan-cardiomyopathy gene panel sequencing and variant calling

We designed a custom pan-cardiomyopathy panel containing 115 genes known to be associated with DCM, HCM, and ARVC, as well as to channelopathies (S2 Table). The custom gene panel, encompassing all exons of each gene and splicing sites, was based on the Illumina strategy of targeted enrichment of these regions. Using this custom panel, we performed genetic screening of the 38 unrelated patients described above, obtaining an average of two millions of 150-bp paired-end reads per sample.

A total of 3286 missense variants, 18 nonsense variants, 23 splicing site variants, 29 in-frame and 6 frameshift insertion/deletions were identified using the ODESSA pipeline, providing an average of 85 variants per DCM patient, 89 per HCM patient, and 92 per ARCV patient (S4 Table). Overall, the mean read depth of coverage for these selected variants was 222X (range, 12–565X). Variants that fulfilled the inclusion criteria described in the Materials and Methods were annotated as rare variants and classified using standard nomenclature (S1 Fig).

### Variant assessment

In total, we detected 142 rare variants in 40 genes, of which 137 variants had a heterozygous status and 5 variants on the X chromosome had a hemizygous status. Thirty-three (23,2%) of these 142 rare variants were absent from both dbSNP and gnomAD (S5 Table). One hundred and twenty-four rare variants were missense variants (87%), while 5 were frameshift insertion/deletion variants, 3 in-frame insertion/deletion variants, 8 nonsense variants, and 2 involved a splicing site (S6 Table). The *TTN* gene had the largest number of rare variants (42 variants), followed by *OBSCN* (16 variants) and *RYR2* (6 variants).

Considering rare variants for each cardiomyopathy type, in ARVC patients, the highest number of rare variants was found in *TTN* and *RYR2* genes (4 variants) followed by *OBSCN* and *PKP2* (3 variants) (S7 Table). For DCM patients, the highest number of rare variants was found in *TTN* gene (26 variants), followed by *OBSCN* (8 variants) and *ANK2* (4 variants) genes (S8 Table). For HCM patients, the highest number of rare variants was found in *TTN* gene (12 variants), followed by *OBSCN* (5 variants) and *MYBPC3* (4 variants) genes (S9 Table).

All patients carried at least one rare variant, while most of them (94.7%) carried more than one rare variant; on average, 3.7 rare variants per patient (range, 1–9) were detected, with DCM patients carrying the highest average number of rare variants (4.5 per patient), followed by HCM (3.6 per patient), and ARVC (2.4 per patient) (S10 Table).

Of the 142 rare variants, 28 were classified as potentially pathogenic, 9 of which already present in ClinVar and/or HGMD databases and/or supported by published data (Table 1 and S11 Table). Fifteen potentially pathogenic variants were missense mutations, 4 were frameshift insertion/deletion mutations, 2 were in-frame insertion/deletion mutations, 5 were nonsense mutations, and 2 affecting a splicing site (Table 1). We excluded from the list of the potentially pathogenic variants, all the *TTN* missense variants indicated as potentially pathogenic by the analysis with the bioinformatics tools because, as recently reported, there is no statistical difference in frequency between cases and controls and their potential pathogenic role can be established only by a segregation analysis in affected families [48, 49]. Regarding the *TTN* truncating variants, we followed the recent evidences that suggest to consider as potentially pathogenic only those variants affecting all TTN isoforms in the A-band region [50, 51]. We identified three *TTN* truncating variants in DCM patients. We considered as potentially pathogenic only the *TTN* truncating variant V16477fs, referred to the N2B isoform (NM_003319) as it maps in the TTN A-band region and was also not present in gnomAD database. The R2490fs variant, referred to the Novex-3 transcript (NM_1333379) and the L24944X variant, referred to the N2B transcript (NM_003319) were not considered potentially pathogenic as they mapped in the I and M-band regions of the TTN isoforms, respectively.

**Table 1:** Potentially pathogenic rare variants detected in our patients.

*Gene*	*Genomic* *Position*	*Transcript*	*Nucleotide*	*Protein*	*dbSNP*	*MAF*	*Cardiac* *Phenotype*	*Supporting evidences*
*ACTC1*	chr15:35084392	NM_005159	c.707C>T	S236F	-	-	HCM	GS, SI, PR, PP2, MT, PC, PP
*AKAP9*	chr7:91726576	NM_005751	c.10303C>T	R3435X	-	-	DCM	MT
*AKAP9*	chr7:91737871	NM_005751	c.11610C>G	Y3870X	rs757753258	4.06x10-6	HCM	MT
*DLG1*	chr3:196812473	NM_004087	c.1915G>A	G639R	rs369412843	8.12x10-6	HCM	GS, SI, PR, PP2, MT, PC, PP
*DMD*	chrX:32456458	NM_004009	c.3959G>A	R1320H	rs768990357	2.24x10-5	ARVC	SI, PR, PP2, MT, PC, PP
*DSP*	chr6:7569522	NM_004415	c.1524dupG	V508fs	-	-	DCM	MT
*DSP*	chr6:7580243	NM_004415	c.3820G>C	A1274P	-	-	DCM	SI, PR, PP2, MT, PP
*LAMP2*	chrX:119576454	NM_013995	c.928G>A	V310I	rs104894858	-	HCM	PP2, MT, PC, PP, CV, HG
*LMNA*	chr1:156100468	NM_170708	c.667_687dup	L140_A146dup	-	-	DCM	REF
*LMNA*	chr1:156106964	NM_170708	c.1549C>T	Q517X	-	-	DCM	MT, PC, PP, HG
*MYBPC3*	chr11: 47371475	NM_000256	c.506-2A>C	-	rs397516057	-	HCM	MT, PC, PP, CV
*MYBPC3*	chr11:47369407	NM_000256	c.821+1G>A	-	rs397516073	2.98x10-5	HCM	MT, PC, PP, CV, HG
*MYBPC3*	chr11:47367758	NM_000256	c.1090G>A	A364T	-	-	HCM	SI, PR, PP2, MT, PC, PP
*MYBPC3*	chr11:47360197	NM_000256	c.2182G>T	E728X	rs397515954	-	HCM	MT, PC, PP, CV
*MYH7*	chr14:23895233	NM_000257	c.2102G>A	G701D	-	-	HCM	SI, PR, PP2, MT, PC, PP
*MYH7*	chr14:23886383	NM_000257	c.4498C>T	R1500W	rs45544633	-	DCM	SI, PR, PP2, MT, CV, HG
*MYH7*	chr14:23884353	NM_000257	c.5410G>A	A1804T	rs730880818	4.06x10-6	DCM	SI, PR, PP2, MT, PC, PP, CV
*NEXN*	chr1:78401657	NM_144573	c.1398_1400delAAT	I467del	-	-	HCM	MT, PC
*OBSCN*	chr1:228400286	NM_052843	c.802G>T	E268X	-	-	HCM	MT, PP
*OBSCN*	chr1:228525823	NM_001098623	c.16979C>T	A5660V	rs191098985	0.0006	DCM	GS, SI, PR, PP2, MT, PC, PP
*OBSCN*	chr1:228527758	NM_001098623	c.17371G>C	A5791P	rs200362121	0.0004	ARVC	SI, PR, PP2, MT, PC, PP
*OBSCN*	chr1:228557681	NM_001098623	c.20006G>A	R6669H	rs373638525	9.4x10-5	DCM	SI, PR, PP2, MT, PP
*PKP2*	chr12:33030842	NM_004572	c.962_972delTCGGCCAGGCG	V321GfsX11	-	-	ARVC	MT
*PKP2*	chr12:32974392	NM_004572	c.2043delT	I681fs	-	-	ARVC	MT,
*RAF1*	chr3:12626632	NM_002880	c.1657A>C	N553H	rs745876012	4.06x10-6	HCM	SI, PR, PP2, MT, PC, PP
*RYR2*	chr1:237802395	NM_001035	c.7009G>C	G2337R	-	-	ARVC	GS, SI, PR, PP2, MT, PC, PP
*TNNT2*	chr1:201334425	NM_001276345	c.305G>A	R102Q	rs121964856	-	HCM	SI, PR, PP2, MT, PC, PP, CV, HG
*TTN*	chr2:179434235	NM_003319	c.49429delG	V16477fs	-	-	DCM	MT, PC, PP

Last revised May 2017. MAF: minor allele frequency in gnomAD; GS: Grantham’s score>100; SI: classified as “Dangerous” by SIFT; PR: classified as “Deleterious” by PROVEAN; PP2: classified as “Problably Damaging” or “Possibly Damaging” by PolyPhen2; MT: classified as “Disease Causing” by Mutation Taster; PC: PhastCons score = 1; PP: PhyloP score>1; CV: classified as “Pathogenic” in ClinVar in association with the same cardiac phenotype; HG: described in HGMD in association with the same cardiac phenotype; REF: published data supporting the variant pathogenicity. Genomic coordinates are referred to the hg19 version of the human genome.

In twelve patients (310DCM, 365DCM, 968DCM, 1584DCM, 1669DCM, 1717DCM, 1838DCM, 1685HCM, 1798HCM, 1665ARVC, 1708ARVC and 1830ARVC), no potentially pathogenic variant was identified, whereas 3 patients (76DCM, 1173HCM and 1776HCM) carried two potentially pathogenic variants (Table 2 and S10 Table).

**Table 2:** Genetic profile of each cardiomyopathic patient carrying potentially pathogenic rare variants.

*Patient*	*Gene*	*Genomic* *Position*	*Transcript*	*Nucleotide*	*Protein*
76DCM					
	***AKAP9***	**chr7:91726576**	**NM_005751**	**c.10303C>T**	**R3435X**
	*DSP*	chr6:7569522	NM_004415	c.1524dupG	V508fs
99DCM					
	*TTN*	chr2:179434235	NM_003319	c.49429delG	V16477fs
682DCM					
	*OBSCN*	chr1:228557681	NM_001098623	c.20006G>A	R6669H
737DCM					
	*LMNA*	chr1:156100468	NM_170708	c.667_687dup	L140_A146dup
1060DCM					
	*DSP*	chr6:7580243	NM_004415	c.3820G>C	A1274P
1329DCM					
	*MYH7*	chr14:23884353	NM_000257	c.5410G>A	A1804T
1718DCM					
	*OBSCN*	chr1:228525823	NM_001098623	c.16979C>T	A5660V
1801DCM					
	*MYH7*	chr14:23886383	NM_000257	c.4498C>T	R1500W
1816DCM					
	*LMNA*	chr1:156106964	NM_170708	c.1549C>T	Q517X
1173HCM					
	*MYBPC3*	chr11:47360197	NM_000256	c.2182G>T	E728X
	*OBSCN*	chr1:228400286	NM_052843	c.802G>T	E268X
1657HCM					
	*MYBPC3*	chr11:47371475	NM_000256	c.506-2A>C	-
1661HCM					
	*MYBPC3*	chr11:47371475	NM_000256	c.506-2A>C	-
1674HCM					
	*NEXN*	chr1:78401657	NM_144573	c.1398_1400delAAT	I467del
1699HCM					
	*MYBPC3*	chr11:47369407	NM_000256	c.821+1G>A	-
1721HCM					
	*RAF1*	chr3:12626632	NM_002880	c.1657A>C	N553H
1739HCM					
	*TNNT2*	chr1:201334425	NM_001276345	c.305G>A	R102Q
1740HCM					
	*LAMP2*	chrX:119576454	NM_013995	c.928G>A	V310I
1741HCM					
	*ACTC1*	chr15:35084392	NM_005159	c.707C>T	S236F
1776HCM					
	***DLG1***	**chr3:196812473**	**NM_004087**	**c.1915G>A**	**G639R**
	*MYBPC3*	chr11:47367758	NM_000256	c.1090G>A	A364T
1832HCM					
	*MYH7*	chr14:23895233	NM_000257	c.2102G>A	G701D
1833HCM					
	***AKAP9***	**chr7:91737871**	**NM_005751**	**c.11610C>G**	**Y3870X**
1662ARVC					
	***OBSCN***	**chr1:228527758**	**NM_001098623**	**c.17371G>C**	**A5791P**
1666ARVC					
	*PKP2*	chr12:32974392	NM_004572	c.2043delT	I681fs
1751ARVC					
	***DMD***	**chrX:32456458**	**NM_004009**	**c.3959G>A**	**R1320H**
1812ARVC					
	*RYR2*	chr1:237802395	NM_001035	c.7009G>C	G2337R
1825ARVC					
	*PKP2*	chr12:33030842	NM_004572	c.962_972del TCGGCCAGGCG	V321GfsX11

New gene-phenotype associations are in bold characters.

### New gene–phenotype associations

**Fig 1:**
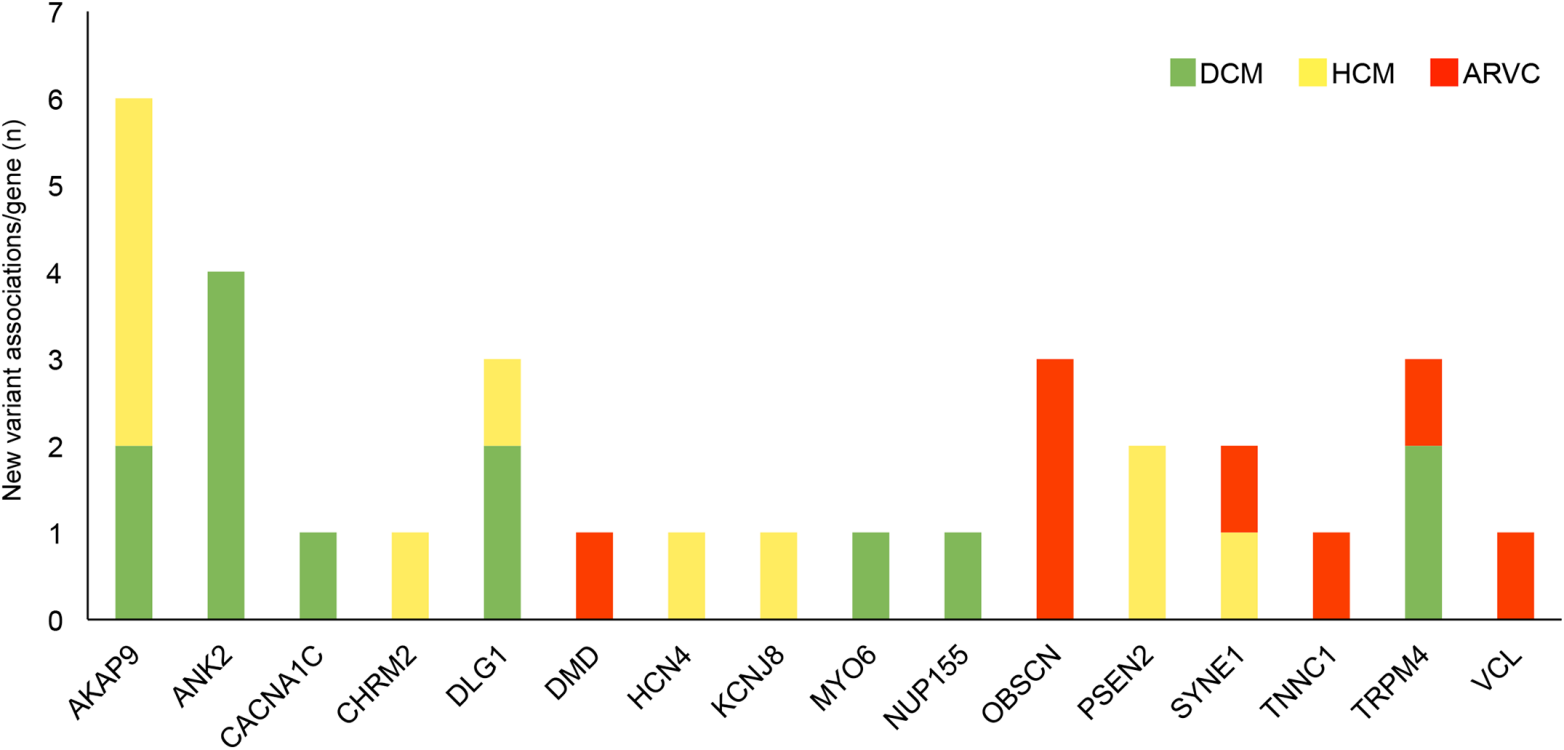
New gene–phenotype associations and number of novel gene rare variants for each cardiomyopathic phenotype.

Most of the rare variants identified in our study showed a well-established association with a specific cardiomyopathic phenotype. In addition, in 23 of 38 patients, we found at least one novel potential gene–phenotype association (Table 2 and S10 Table). As shown in Fig 1, considering the most represented genes, we found six new associations for *AKAP9* gene, four for *ANK2* gene, three for *DLG1*, *OBSCN* and *TRPM4* genes and two for *PSEN2* and *SYNE1* genes. In particular, we focused on potentially pathogenic rare variants to examine the novel gene–phenotype associations (Table 2 and S11 Table). For the ARVC phenotype, we found two potentially pathogenic variants involving *DMD* and *OBSCN* genes, not previously associated with this phenotype. Indeed, we found one novel potentially pathogenic missense variant in *DMD* gene in patient identified as 1751ARVC (DMD_R1320H) and one potentially pathogenic missense variant in *OBSCN* gene.

Regarding the HCM phenotype, we identified two potentially pathogenic variants involving *DLG1* and *AKAP9* genes, not previously associated with this phenotype. In *DLG1* gene, a potentially pathogenic missense variant was found in patient identified as 1776HCM (*DLG1*_G639R); in *AKAP9* gene, a novel potentially pathogenic nonsense variant was found in patient identified as 1833HCM (*AKAP9*_Y3870X).

With regard to DCM, the association between potentially disease-causing variants in *OBSCN* gene and this phenotype was recently described [54]. Our findings were in agreement with this new association, as we found two potentially pathogenic variants in *OBSCN* gene, one in patient identified as 682DCM (*OBSCN*_R6669H) and the other in patient identified as 1718DCM (*OBSCN*_A5660V). Moreover, for DCM phenotype, we identified one potentially pathogenic variant involving the *AKAP9* gene, not previously associated with DCM, in patient identified as 76DCM (*AKAP9*_R3435X).

## Discussion

The technology of NGS is now emerging as a powerful approach to comprehensively explore genetic mutations in a wide range of human pathologies [36, 55]. In this study, we used targeted resequencing to identify novel genetic variations associated with three inherited cardiomyopathies, namely HCM, DCM, and ARVC, which are common causes of mortality before the fifth decade of life. These inherited cardiomyopathies are genetically heterogeneous and are associated with rare mutations in a large number of genes, many of which overlap among the phenotypes. Moreover, considerable phenotypic and genetic overlaps between cardiomyopathies and arrhythmic syndromes have been documented [22–29]. Considering these overlaps, our study aimed to look for new potential gene–phenotype associations with a view to expand the mutational spectrum underlying cardiomyopathies, with possible implications for better diagnosis and clinical management. We designed a custom gene panel including 115 genes, known to be associated with the three cardiomyopathic phenotypes and/or arrhythmic syndromes. Using this panel, we performed molecular screening of a cohort of 38 unrelated patients affected by cardiomyopathies (16 DCM, 14 HCM, and 8 ARVC). Overall, our analysis identified a total of 142 rare variants in 40 genes and confirmed 11 rare variants (*ACTC1*_S236F, *LAMP2*_V310I, *LMNA*_Leu140-Ala146dup, *LMNA*_Q517X, *MYBPC3*_A364T, *MYBPC3*_c.821+1G>A, *MYBPC3*_E728X, *MYH7*_A1763T, *MYH7*_G701D, *MYL2*_E134A, and *TNNT2*_R102Q) which we previously identified in some of our patients by Sanger sequencing [52, 56], thereby demonstrating that our approach, that combined NGS and different *in silico* prediction tools, was both reliable and sensitive at identifying rare variants. Interestingly, for 75 genes in our panel, no rare variant was identified, suggesting that some gene–phenotype associations reported in the literature (on which our gene panel was based) are restricted to unique or few cases.

Our results confirm previous data indicating that cardiomyopathic patients could carry multiple specific genetic variants [33, 34, 57]. In fact, we found on average more than three rare variants per proband, with DCM patients carrying the highest average number of rare variants, followed by HCM and ARVC patients. Furthermore, since our subjects were selected on the basis of phenotype severity and family history of cardiomyopathy and/or SD, our results support the evidence that multiple mutations are likely to elicit a more severe phenotype. Indeed, previous studies suggested that patients carrying multiple mutations are more likely to develop the disease at an early age and with more severe clinical features, as poor prognoses are associated with compound or double heterozygotes [33, 34].

Titin is the largest described human protein, constituting one of the most abundant types of filaments in both cardiac and skeletal muscle and plays a central role in sarcomere organization. *TTN* has been recognized as the main disease-causing gene in DCM and is also involved in the pathogenesis of HCM and ARVC [17, 49, 58]. Our study confirmed the high prevalence of *TTN* rare variants in cardiomyopathies, most of which are missense. *TTN* missense variants are very common and often frequently benign and it was reported that *TTN* missense variants bioinformatically classified as “severe” did not associate with differences in clinical phenotypes in DCM patients, so arguing that many *TTN* missense variants are not disease-causing variants [48]. For these reasons, many *TTN* missense variants identified in our study were not classified as potentially pathogenic, despite the results of predictors’ analysis. Regarding *TTN* truncating variants, recent studies supported that not all these variants are equals in terms of clinical relevance. In particular, the *TTN* truncating variants with the highest probability of pathogenicity are those affecting TTN isoforms in the A-band region [50, 51]. In our study, we identified three *TTN* truncating variants in DCM patients: V16477fs (referred to NM_003319 transcript), L24944X (referred to NM_003319 transcript) and R2490fs (referred to NM_133379 transcript). According to recent evidences, we considered as potentially pathogenic, with a possible clinical relevance, only the V16477fs variant, as it maps in the A-band region and affects all *TTN* transcripts. However, it cannot be ruled out that multiple variants (missense and truncating) on the TTN protein may have a cumulative effect on a cardiomyopathic phenotype.

**Fig 2:**
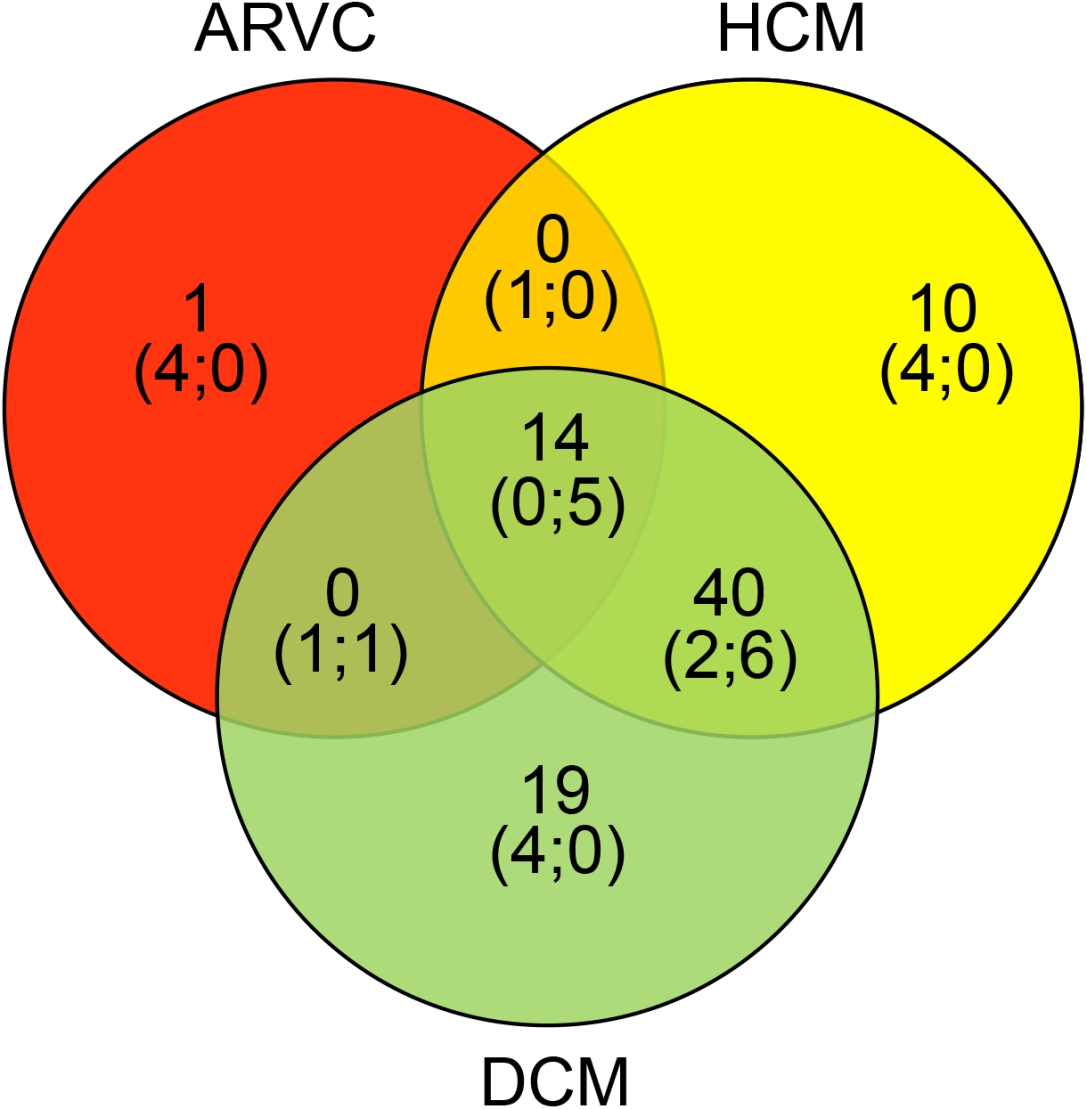
Distribution of specific and overlapping genes carrying rare variants identified in this study, among the three cardiomyopathic phenotypes.

In this study, we extended the number of overlapping genes among cardiomyopathies and also between cardiomyopathies and arrhythmic syndromes, identifying novel potential gene-phenotype associations (Fig 1 and S2 Table). As reported in Fig 2, which shows the distribution of specific and overlapping genes carrying rare variants identified in this study, among the three cardiomyopathic phenotypes, we detected five genes (*DMD*, *OBSCN*, *SYNE1*, *TNNC1*, *VCL*) carrying rare variants in the three phenotypes, six genes (*AKAP9*, *CACNA1C*, *CHRM2*, *DLG1*, *MYO6*, *PSEN2*) carrying rare variants in HCM and DCM, and one gene (*TRPM4*) carrying rare variants in ARVC and DCM. As several patients carried variants in genes not previously related to their phenotype, the use of our “pan-cardiomyopathy panel” may prove beneficial to identify the novel potential gene-phenotype associations. In fact, a phenotype-specific targeted gene testing [30] might miss to identify several variants in cardiomyopathic patients. Conversely, a comprehensive gene testing could enhance the diagnostic and prognostic implications of genetic screening by not only allowing an early diagnosis of inherited cardiomyopathic forms but also by contributing to a more accurate prognostic stratification and patient management [31].

*OBSCN* variants were firstly described in HCM patients [59] and, more recently, *OBSCN* variants associated with DCM and Left Ventricular Noncompaction patients were reported [54, 60]. Our results are in agreement with the association between *OBSCN* gene variants and DCM phenotype, since we found eight different rare variants in 8 DCM patients. When our analysis was restricted to potentially pathogenic variants, the association between *OBSCN* variants and DCM remained, because two potentially pathogenic variants, *OBSCN*_R6669H and *OBSCN*_A5660V, were detected in patients identified as 682DCM and 1718DCM, respectively (Table 2). Furthermore, for the first time to our knowledge a possible association between *OBSCN* gene and the ARVC phenotype is evidenced. In fact, we found three different *OBSCN* rare variants in two out of eight ARVC probands. One of these variants, *OBSCN*_A5791P was classified as potentially pathogenic (Table 1 and S11 Table) and was detected in patient identified as 1662ARVC (Table 2). A possible pathogenic role for *OBSCN* variants detected in some of our DCM and ARVC patients is supported by the fact that these variants involve OBSCN domains having functional and/or structural importance and affect highly conserved amino acid regions across species. Obscurin is a member of a family of giant proteins that is expressed in striated muscle and interacts with titin, myomesin and small ankyrin-1 and has been proposed to be a structural protein linking the M-line of the sarcomere to the sarcoplasmatic reticulum [61, 62]. Different obscurin isoforms are known: obscurin A (~720 kDa); obscurin B (~870 kDa), which is similar to obscurin A but lacking the nonmodular COOH-terminal region and including two Ser/Thr kinase domains, called SK2 and SK1; and two smaller obscurin isoforms, one containing a full-length SK1 and a partial SK2 and the other containing only the SK1 domain. The expression of these obscurin isoforms differs; while giant obscurins A and B are expressed in higher amounts in skeletal muscle, the tandem and single kinase isoforms are largely present in cardiac muscle [62]. Variant *OBSCN*_R6669H involves the SK1 domain, and variant *OBSCN*_A5791P falls in a functionally important domain of obscurin, the tandem Rho-guanidine nucleotide exchange factor (RhoGEF) present in Rho/Rac/Cdc42-like GTPases, and thus these variants may affect obscurin cell signaling pathways. Variant *OBSCN*_A5660V is present in the src homology-3 (SH3)-conserved domain, a protein interaction domain. Therefore, OBSCN appears to be a good candidate gene for both DCM and ARVC phenotypes. Recent data supported *OBSCN* mutations as a significant causal factor for DCM pathogenesis, reporting that disease-related *OBSCN* mutations cause haploinsufficiency that accounts for the development of the DCM phenotype [54]. Moreover, it was suggested that *OBSCN* mutations could act alone or in concert with other mutations, contributing to cause DCM [54, 63]. Our data are in agreement with this last hypothesis, since patients carrying a potentially pathogenic variant in the *OBSCN* gene also carried other rare variants in different genes, such as *PKP2*, or *TTN*, suggesting a possible cumulative effect on cardiomyopathic phenotype.

For the ARVC phenotype, we also found an association with the *DMD* gene. Mutations in the *DMD* gene, encoding for dystrophin, are responsible for X-linked DCM and myopathies [64–66]. ARVC patients were found to be affected by myofibrillar myopathy [67, 68], facio-scapulo-humeral muscular dystrophy [69], and myotonic dystrophy type 1 [70]. An ARVC-like phenotype was described in desmin-related myopathy [71] and myotonic dystrophy type 1 [72]. In addition, some of the genes recently found to be associated with ARVC are also responsible for myopathies/muscular dystrophies, such as *DES* [73], *LDB3* [74], and *TTN* [75]. Moreover, both phenotypic and genetic overlaps between DCM and ARVC were observed [52, 75–77].

We identified the *DMD*_R1320H variant in the patient identified as 1751ARVC, who was diagnosed with ARVC and associated LV systolic dysfunction at the age of 42 years. The novel variant is localized in the spectrin domain number 9 of dystrophin and is predicted to be pathogenic *in silico* because it is believed to affect two *DMD* transcripts. Mutations in dystrophin affecting the plasma membrane and causing myofiber loss could lead to the pathologic substrate favoring myocardial atrophy and fibrofatty replacement, which are the main pathogenetic mechanisms described in ARVC [78]. However, more evidences are necessary to consider *DMD* as a new gene associated with the ARVC phenotype.

Although hereditary cardiomyopathies and arrhythmic syndromes are considered distinct groups of genetic disorders, a considerable phenotypic overlap between these two apparently distinct cardiac disorders has been documented. In fact, the clinical cardiomyopathic picture often includes a wide range of features related to arrhythmias and conduction abnormalities; conversely, ion channel defects linked to arrhythmic syndromes may be associated with morphological changes and structural abnormalities that characterize some types of cardiomyopathy [5, 24–29, 79]. In a recent study that screened HCM probands for both sarcomere and non-sarcomere protein gene mutations, *ANK2*, *PLN*, and *SCN5A* gene variants were suggested to have modifying effects on HCM phenotypic expression [26]. Moreover, novel associations between three genes (*KCNQ1*, *KCNH2*, and *KCNE2*) associated with Long QT syndrome and HCM phenotype were proposed [26]. As the main goal of our study was to look for new gene–phenotype associations, we included in our custom panel some genes associated with inherited arrhythmic syndromes in order to support the evidence that genes implicated in channelopathies could modify the clinical characteristics and severity of cardiomyopathic phenotypes. We found novel potential gene–phenotype associations between genes typically related to arrhythmic syndromes (*AKAP9*, *ANK2*, *CACNA1C*, *DLG1*, *HCN4*, *KCNJ8*, *NUP155* and *TRPM4* genes) and the cardiomyopathic phenotypes discussed in our study (S2, S9 and S11 Tables). In particular, focusing on the potentially pathogenic variants, we detected new associations between *AKAP9* variants and both DCM and HCM and between *DLG1* variant and HCM (Table 2 and S11 Table).

A novel association between *AKAP9* gene and DCM was observed in patient identified as 76DCM. This patient demonstrated an early-onset (15 years) DCM phenotype and presented with a history of palpitations, syncope on effort, and evidence of frequent multifocal premature ventricular complexes, non-sustained VT episodes, and atrioventricular conduction abnormalities recorded on Holter monitoring. He received an ICD. During follow-up, he suffered from recurrent episodes of sustained VT and VF discontinued by ICD shocks. This patient carried two potentially pathogenic variants: *DSP*_V508fs variants and *AKAP9*_R3435X (Table 2 and S11 Table). *DSP*_V508fs is a novel variant, predicted to produce a truncated protein and to reduce DSP expression. *AKAP9*_R3435X is predicted to produce a truncated protein from three different *AKAP9* transcripts, thus suggesting either a dominant negative mechanism or a nonsense-mediated mRNA decay mechanism. We suppose that the *DSP*_V508fs variant is the primary disease-causing mutant since the *DSP* gene has been demonstrated to be associated with DCM (OMIM: 615821) and biventricular arrhythmogenic cardiomyopathy (OMIM: 607450) and that *AKAP9* may be a modifying gene causing severe ventricular tachyarrhythmias, such as those occurring in the patient identified as 76DCM. This hypothesis is supported by the evidence that *AKAP9* has been considered as a genetic modifier of congenital LQTS, increasing cardiac risk and disease severity in *AKAP9* mutation carriers [80].

Another potentially pathogenic variant (*AKAP9*_Y3870X) is present in the patient identified as 1833HCM, who also carries the *MYL2*_E134A variant (S10 Table). The *MYL2* variant may have a pathogenic role in 1833HCM patient, as previously described for the same phenotype [81], although it is classified as VUS in ClinVar database. The *AKAP9*_Y3870X variant may act as a disease severity modifier, even though the stop codon introduced by this mutation is located in the C-terminal part of the protein; so, its effect should be analyzed in functional studies and/or segregation in families.

*DLG1* encodes the synapse-associated protein 97 protein (SAP97), a protein belonging to the membrane-associated guanylate kinase (MAGUK) family of proteins that are important for localization and organization of ion channels. SAP97 interacts with and modulates cardiac Kv4.3 channels that account for a large part of the transient outward potassium current (Ito) [82], but also binds and regulates the expression of NaV1.5, the main cardiac inward sodium channel [83]. Both channels are implicated in Brugada syndrome (BRGDA1, OMIM: 601144; BRGDA9, OMIM: 616399), and consequently a *DLG1* variant may confer susceptibility to arrhythmias as demonstrated by a putative *DLG1* pathogenic mutation in a patient affected by this syndrome [84]. We found the potentially pathogenic variant *DLG1*_G639R in patient identified as 1776HCM, also carrying the potentially pathogenic variant *MYBPC3*_A364T (Table 2, S10 and S11 Tables). The *MYBPC3*_A364T variant may be considered pathogenic for this patient, as *MYBPC3* is one of the main genes associated with HCM. The *DLG1*_G639R variant involves the DLG1 SH3 domain, which mediates the assembly of specific protein complexes via binding to proline-rich peptides. It is conceivable that this mutation could affect DLG1-Kv4.3 or DLG1-NaV1.5 interactions, modulating their functions and favoring susceptibility for arrhythmias.

Our study demonstrates that a targeted resequencing approach can provide a relevant support for a genetic diagnosis in cardiomyopathic patients. However, in many patients, in particular, those affected by DCM, it was not possible to obtain a clear genetic diagnosis on the basis of our gene panel, thus suggesting a greater genetic heterogeneity in this cardiomyopathy respect to the others and/or a considerable role of modifier genes in determining this phenotype. Gene discovery by whole exome or genome sequencing would be required for those probands that are still without a molecular diagnosis. However, the application of this approach would make the interpretation of genetic screening results difficult for clinical purposes due to the large amount of sequencing data that need to be analysed.

We are aware that our study would need to be supported by evidences in favor of a pathogenic role of the variants reported here (e.g., lack of segregation analysis due to sporadic cardiomyopathy, and/or unavailability and/or small number of relatives available for genetic investigations and lack of functional studies) [85], not allowing us to assess the clinical relevance of the identified variants. Segregation analysis together with functional studies may concur to establish a more definite diagnosis, allowing the findings to be interpreted in the context of a clinical setting. However, families with multiple affected individuals are a rare occurrence and often a novel variant is present in a single individual with only few or no other clinically affected relatives. Consequently, many novel NGS variants are classified as VUS and cannot represent a conclusive test result at present, as recently highlighted [85].

On the other hand, we believe that the rigorous approaches used in this study to identify the cardiomyopathies associated variants (e.g. recruitment of patients with more severe phenotypes and family history of cardiomyopathy and/or sudden death, selection of variants using a MAF lower than the frequency of each cardiomyopathy, application of different *in silico* prediction tools to select the potentially pathogenic variants) reliably support our hypothesis that many of them may contribute to cardiomyopathy development and/or its progression. In particular, in agreement with results obtained by Marston et al. [54], mutations in the *OBSCN* gene could be considered a significant causal factor, alone or in concert with other mutations, for DCM and ARVC pathogenesis. Furthermore, variants in *AKAP9* and *DLG1* genes could act as genetic modifiers of arrhythmic risk and phenotype severity in cardiomyopathies. In addition, variants of unknown pathogenicity, sometimes complicating the clinical picture, may contribute to the disease development and/or may have modifier effects. It is possible to hypothesize that the genetic component underlying cardiomyopathic phenotypes and/or modifying disease phenotype severity could be explained by the cumulative functional effects of coding and non-coding DNA variants.

To conclude, this work highlights the challenges faced in the diagnosis of cardiomyopathies. NGS is a highly accurate and reproducible approach for routine molecular screening of patients with cardiomyopathies, but the identification of genetic defects in the proband is not sufficient in isolation for clinical decision-making or genetic counselling.

Finally, our data support the remarkable contribution that a targeted NGS approach could offer to genetic overlap knowledge between different cardiomyopathies, expanding previous results and ameliorating molecular diagnosis.

In the brackets, we report the number of the new potential gene-phenotype associations (number on the left) and the number of the overlapping genes (number on the right) detected in this study (see also underlined associations in S2 Table).

## Supporting information

S1 Fig Variant classification. **A. Missense, stop-gain, stop-loss and splice site variant analysis.** Single nucleotide variant calls supported by at least 10 reads and with a minor allele frequency ≤ 0.004, ≤ 0.002 and ≤ 0.0005 for DCM, HCM and ARVC patients respectively in variant databases, were considered. Missense variants were analyzed using Mutation taster, SIFT, Provean and Polyphen-2 and retained if predicted to be “disease causing” by Mutation Taster, “damaging” by SIFT, “deleterious” by Provean and “probably” or “possibly damaging” by Polyphen-2 and considered Potentially Pathogenic missense variants if not classified as VUS, Likely Benign or Benign in ClinVar database. Stop-gain, stop-loss and splicing site variants were analyzed using Mutation Taster and considered Potentially Pathogenic radical variants if predicted to be “disease causing” and if not classified as VUS, Likely Benign or Benign in ClinVar database.
**B. Insertion/deletion variant analysis.** Frameshift and in-frame insertion/deletion variants supported by at least 10 reads with a minor allele frequency ≤ 0.004, ≤ 0.002 and ≤ 0.0005 for DCM, HCM and ARVC patients respectively in variant databases, were considered and classified as rare radical variants. Radical variants were analyzed using Mutation Taster and considered Potentially Pathogenic if predicted to be “disease causing” and if not classified as VUS, Likely Benign or Benign in ClinVar database.
Missense and radical variants were also considered as Potentially Pathogenic variants, independently from the results obtained with the above predictors, if classified as “Pathogenic” or “Likely Pathogenic” in published data and/or in ClinVar or HGMD database, in association with cardiomyopathies. *TTN* missense variants were not considered potentially pathogenic independently from predictors’ responses. *TTN* truncating variants were considered potentially pathogenic if they affected all TTN isoforms in the A-band region.
(TIF)

S1 Table Clinical features of patients. (DOC)

S2 Table List of genes included in our panel. (DOC)

S3 Table Accession numbers of sequencing data for each patient included in this study submitted to “The European Genome-phenome Archive”. (DOC)

S4 Table Number of total variants detected in all patients, according to variant classification. (DOC)

S5 Table Rare variants detected in our patients. (DOC)

S6 Table List of the mutated genes and number and type of rare variants detected in all patients. (DOC)

S7 Table List of the mutated genes and number and type of rare variants detected in ARVC patients. (DOC)

S8 Table List of the mutated genes and number and type of rare variants detected in DCM patients. (DOC)

S9 Table List of the mutated genes and number and type of rare variants detected in HCM patients. (DOC)

S10 Table Rare variants detected in each patient. (DOC)

S11 Table Potentially pathogenic rare variants according to *in silico* prediction tools. (DOC)
