# Supplementary figures and images for: Targeted next-generation sequencing detects novel gene–phenotype associations and expands the mutational spectrum in cardiomyopathies
Source: PLoS One. 2017 Jul 27;12(7):e0181842. doi: 10.1371/journal.pone.0181842 (PMC5531468; doi:10.1371/journal.pone.0181842)

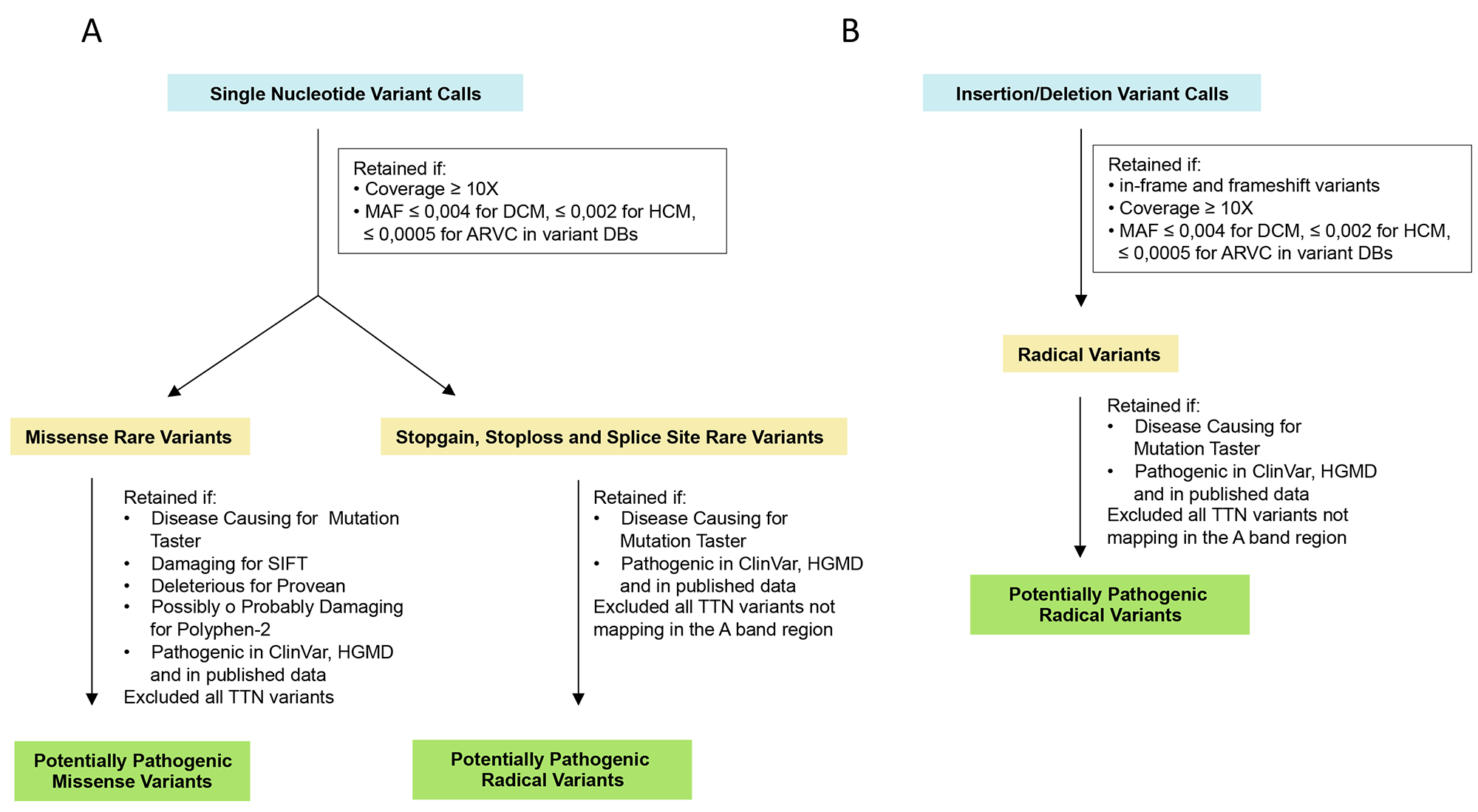

Supplement: S1 Fig — A. Missense, stop-gain, stop-loss and splice site variant analysis. Single nucleotide variant calls supported by at least 10 reads and with a minor allele frequency ≤ 0.004, ≤ 0.002 and ≤ 0.0005 for DCM, HCM and ARVC patients respectively in variant databases, were considered. Missense variants were analyzed using Mutation taster, SIFT, Provean and Polyphen-2 and retained if predicted to be “disease causing” by Mutation Taster, “damaging” by SIFT, “deleterious” by Provean and “probably” or “possibly damaging” by Polyphen-2 and considered Potentially Pathogenic missense variants if not classified as VUS, Likely Benign or Benign in ClinVar database. Stop-gain, stop-loss and splicing site variants were analyzed using Mutation Taster and considered Potentially Pathogenic radical variants if predicted to be “disease causing” and if not classified as VUS, Likely Benign or Benign in ClinVar database. B. Insertion/deletion variant analysis. Frameshift and in-frame insertion/deletion variants supported by at least 10 reads with a minor allele frequency ≤ 0.004, ≤ 0.002 and ≤ 0.0005 for DCM, HCM and ARVC patients respectively in variant databases, were considered and classified as rare radical variants. Radical variants were analyzed using Mutation Taster and considered Potentially Pathogenic if predicted to be “disease causing” and if not classified as VUS, Likely Benign or Benign in ClinVar database. Missense and radical variants were also considered as Potentially Pathogenic variants, independently from the results obtained with the above predictors, if classified as “Pathogenic” or “Likely Pathogenic” in published data and/or in ClinVar or HGMD database, in association with cardiomyopathies. TTN missense variants were not considered potentially pathogenic independently from predictors’ responses. TTN truncating variants were considered potentially pathogenic if they affected all TTN isoforms in the A-band region. (TIF) [file pone.0181842.s001.tif]
