# Supplementary material for: Targeted next-generation sequencing detects novel gene–phenotype associations and expands the mutational spectrum in cardiomyopathies
Source: PLoS One. 2017 Jul 27;12(7):e0181842. doi: 10.1371/journal.pone.0181842 (PMC5531468; doi:10.1371/journal.pone.0181842)
Supplement: S1 Table — (DOC) [file pone.0181842.s002.doc]

**S1 Table. Clinical features of patients.**

| **Patient ID** | **CMP type** | **Presenting symptoms** | **Phenotype** | **Clinically affected relatives and/or died suddenly*** |
| --- | --- | --- | --- | --- |
| **76DCM** | DCM | Palpitations, syncope on effort | 2D echo: LVEDD 60 mm, LVEF 29%, RV dilatation, mild MVR, mild TVR; biventricular enlargement at ventriculography; I°-II° AVB; FVE; NSVT; ICD implantation; recurrent episodes of SVT and VF discontinued by ICD shocks. | 0 |
| **99DCM** | DCM | Left arm pain | 2D echo: LVEDD 57 mm, LVEF 35%, mild MVR; LVBBB. | 1 (1) |
| **310DCM** | DCM | Dyspnea, chest pain | 2D echo: LVEDD 70 mm, LVEF 20%, mild MVR; NSVT; ICD implantation. | 1 (0) |
| **365DCM** | DCM | Palpitations, syncope | 2D echo: LVEDD 60 mm, LVEF 35%, mild MVR; paroxysmal AF; FVE; NSVT; ICD implantation; recurrent episodes of SVT discontinued by ICD; VT transcatheter ablation. | 0 |
| **682DCM** | DCM | Asymptomatic | 2D echo: LVEDD 63 mm, LVEF 40%, mild MVR. | 2 (2) |
| **737DCM** | DCM | Palpitations and dyspnea, followed by syncope on effort | 2D echo: LVEDD 59 mm, LVEF 40%, moderate MVR; II° AVB; FVE; NSVT; SVT; ICD implantation; unsuccessful VT transcatheter ablation and heart transplantation for both SVT recurrences in storm and subsequent LV function deterioration (LVEF 30%). | 28 (13) |
| **968DCM** | DCM | Asymptomatic | 2D echo: LVEDD 57 mm, LVEF 45%; FVE. | 4 (3) |
| **1060DCM** | DCM | Chest pain | 2D echo: LVEDD 60 mm, LVEF 35%, mild-moderate MVR; FVE; NSVT. | 2 (2) |
| **1329DCM** | DCM | Dyspnea, chest pain, fatigue, palpitations | 2D echo: LVEDD 58 mm, LVEF 35%; received an ICD. | 0 |
| **1584DCM** | DCM | Asymptomatic | 2D echo: LVEDD 61 mm, LVEF 30%, mild MVR; FVE; NSVT. | 3 (1) |
| **1669DCM** | DCM | Resuscitated cardiac arrest due to VF | 2D echo: LVEDD 57 mm, LVEF 35%, mild MVR; LVBBB; FVE. ICD implantation. | 0 |
| **1717DCM** | DCM | Dyspnea, asthenia | 2D echo: LVEDD 81 mm, LVEF 25%, severe MVR; LVBBB; III°AVB; dual chamber PM implantation and upgrading to biventricular PM due to congestive heart failure; permanent AF; subsequent upgrading to biventricular ICD because of syncope secondary to SVT; appropriate ICD interventions for recurrent SVT episodes; repeated hospitalizations for worsening HF; death from refractory heart failure. | 1 (1) |
| **1718DCM** | DCM | Palpitations | 2D echo: LVEDD 56 mm, LVEF 35%, moderate MVR; FVE; NSVT; persistent AF; ICD implantation. | 0 |
| **1801DCM** | DCM | Asthenia, dyspnea | 2D echo: LVEDD 67 mm, LVEF 25%, moderate-severe MVR. | 2 (2) |
| **1816DCM** | DCM | Left arm pain | 2D echo: LVEDD 62 mm, LVEF 49%, mild-moderate MVR; I°-II° AVB; permanent AF; FVE; NSVT; ICD implantation; patient currently awaiting cardiac transplantation due to LV function deterioration (LVEDD 64 mm; LVEF 25%). | 1 (0) |
| **1838DCM** | DCM | Palpitations | 2D echo: LVEDD 65 mm, LVEF 35%, mild MVR; FVE; ICD implantation. | 1(0) |
| **1173HCM** | HCM | Dyspnea, palpitations | NOHCM (IVS thickness: 23 mm), mild MVR, moderate-severe AVR; FVE; NSVT; ICD implantation. LV function deterioration (LVDD 65 mm, LVEF: 23%). Patient currently awaiting cardiac transplantation. | 2 (2) |
| **1657HCM** | HCM | Asymptomatic | OHCM (IVS thickness 28 mm, LVOT gradient 94 mmHg); NSVT; successful persistent AF ablation; ICD implantation. | 5 (2) |
| **1661HCM** | HCM | Chest pain, palpitations, dizziness | OHCM (IVS thickness 30 mm, LVOT gradient 35 mmHg), moderate MVR; NSVT; ICD implantation; appropriate intervention of ICD on SVT. | 4 (3) |
| **1674HCM** | HCM | Palpitations, dizziness, asthenia | OHCM (IVS thickness 30 mm, LVOT gradient 60 mmHg), mild MVR; FVE; NSVT; ICD implantation. | 0 |
| **1685HCM** | HCM | Chest pain, dyspnea, palpitations, dizziness | OHCM (IVS thickness 25 mm, LVOT gradient 30 mmHg at rest and 131 mm Hg during exercise stress echocardiography), mild MVR; NSVT; syncope; ICD implantation; ventricular septal myectomy. | 2 (2) |
| **1699HCM** | HCM | Dyspnea, palpitations | NOHCM (IVS thickness 29 mm), moderate MVR; NSVT; syncope; ICD implantation; appropriate intervention of ICD on VF. | 1 (1) |
| **1721HCM** | HCM | Resuscitated cardiac arrest due to VF | NOHCM (IVS thickness 15 mm); ICD implantation; appropriate intervention of ICD on VF. | 0 |
| **1739HCM** | HCM | Palpitations, fatigue | NOHCM (IVS thickness 26 mm). ICD implantation. | 7 (2) |
| **1740HCM** | HCM | Dyspnea, palpitations | NOHCM (IVS thickness 17 mm), mild MVR; WPW syndrome; ICD implantation. During the seventh month of pregnancy, the patient developed LV systolic dysfunction (LVEF 30%). Postpartum severe systolic dysfunction persisted, despite optimized medical treatment, and the patient was directed to cardiac transplant clinical and instrumental evaluations. During follow-up, seven episodes of paroxysmal AF were recorded in the arrhythmia register of ICD and one episode of SVT, interrupted by ICD anti-tachycardia pacing. | 1 (0) |
| **1741HCM** | HCM | Palpitations, dizziness | NOHCM (IVS thickness 21 mm), mild MVR, LV systolic dysfunction (LVEF 45%); NSVT; ICD implantation, appropriate intervention of ICD on VF. | 0 |
| **1776HCM** | HCM | Dyspnea, chest pain, palpitations | OHCM (IVS thickness 22 mm, LVOT gradient 128 mmHg), mild MVR; NSVT. | 1 (1) |
| **1798HCM** | HCM | Chest pain, palpitations, dyspnea | OHCM (IVS thickness 24 mm, LVOT gradient 183 mmHg), moderate-severe MVR. Ventricular septal myectomy, and ICD implantation. | 0 |
| **1832HCM** | HCM | Palpitations, syncope | OHCM (IVS thickness 20 mm, LVOT gradient: 32 mmHg), mild MVR and AVR; NSVT; persistent AF. ICD implantation. | 4 (2) |
| **1833HCM** | HCM | Chest pain | NOHCM (IVS thickness 22 mm). ICD implantation. | 1 (1) |
| **1662ARVC** | ARVC | Palpitations | ECG: inverted T waves in right precordial leads (V1, V2, and V3); 2D echo: dilated RV (RVOT 35 mm), moderate TVR; RV angiography: dyskinetic areas with bulging at the RV free wall; FVE; ICD implantation, appropriate intervention of ICD on ventricular tachyarrhythmia. | 4 (1) |
| **1665ARVC** | ARVC | Syncope | 2D echo: dilated RV (RVOT 36 mm), mild TVR; RV angiography: dyskinetic areas with bulging at the RV free wall; sinus node dysfunction; FVE; NSVT with LBBB morphology; ICD implantation; paroxysmal AF transcatheter ablation. | 3 (2) |
| **1666ARVC** | ARVC | Palpitations | ECG: inverted T waves in precordial leads (from V1 to V6); 2D echo: dilated RV (RVOT 39 mm), RV apex and inferior wall akinesia, RV free wall dyskinesia, mild-moderate TVR, mild MVR; RV angiography: dilated RV, dyskinetic areas of the RV inferior and free walls; FVE of LBBB morphology; NSVT; SVT; ICD implantation; ICD shock on SVT recurrences; repeated SVT transcatheter ablation. | 0 |
| **1708ARVC** | ARVC | Chest pain | ECG: complete RBBB, inverted T waves (from V1 to V6); 2D echo: dilated RV (RVOT 50 mm); LV systolic dysfunction (LVEF 40%), severe TVR, mild MVR; RV angiography: severe dilatation and ipokinesia of the RV, dyskinetic areas with diastolic bulging at the RV free wall; LV angiography: LV impairment (LVEF 40%); FVE; NSVT; ICD implantation. | 1 (0) |
| **1751ARVC** | ARVC | Palpitations, asthenia | 2D echo: dilated RV (RVOT 36 mm), LV systolic dysfunction (LVEF 42%), mild TVR, mild MVR; RV angiography: dyskinetic areas with bulging at the RV free wall; LV angiography: global LV hypokinesia (LVEF 42%); FVE; NSVT of LBBB morphology; ICD implantation; episode of SVT discontinued by ICD antitachycardia pacing. | 2 (1) |
| **1812ARVC** | ARVC | Resuscitated cardiac arrest due to VF | 2D echo: dilated RV (RVOT 35 mm), moderate TVR; NSVT; RV angiography: dyskinetic areas with bulging at the RV free wall; ICD implantation; appropriate ICD shocks on SVT and VF. | 8 (0) |
| **1825ARVC** | ARVC | Palpitations, syncope on effort | ECG: inverted T waves from V1 to V5; 2D echo: dilated RV (RVOT 45 mm); RV angiography: dilated RV; IVS and RV apex dyskinetic areas; FVE of LBBB morphology; NSVT; SVT; ICD implantation. | 4 (0) |
| **1830ARVC** | ARVC | Resuscitated cardiac arrest due to VF | ECG: inverted T waves from V1 to V3; 2D echo: dilated RV (RVOT 49 mm); CMR: dyskinetic areas at the free and inferior walls of the RV; I° AVB; FVE; NSVT; paroxysmal AF; ICD implantation. | 4 (3) |

AF = atrial fibrillation; AVR = aortic valve regurgitation; ARVC = arrhythmogenic right ventricular cardiomyopathy; AVB = atrioventricular block; AVR = aortic valve regurgitation; CHF = chronic heart failure; CMP = Cardiomyopathy; CMR = cardiac magnetic resonance imaging; DCM = dilated cardiomyopathy; Dx = diagnosis; 2D echo = echocardiography; F = female; FVE = frequent ventricular extrasystoles (>1000/24 h); HCM = hypertrophic cardiomyopathy; ICD = implantable cardioverter-defibrillator; IVS = interventricular septum; LBBB = left bundle-branch block; LV = left ventricle; LVEDD = left ventricular end-diastolic diameter; LVEF = left ventricular ejection fraction; M = male; MVR = mitral valve regurgitation; LVOT = left ventricular outflow tract; NOHCM = non-obstructive hypertrophic cardiomyopathy; NSVT = non-sustained ventricular tachycardia; OHCM = obstructive hypertrophic cardiomyopathy; PM = pacemaker; RBBB = right bundle-branch block; RV = right ventricle; RVOT = right ventricular outflow tract; SVT = sustained ventricular tachycardia; TVR = tricuspid valve regurgitation; VF = ventricular fibrillation; WPW = Wolff–Parkinson–White syndrome.

* The number of living affected family members is given in parentheses.
