# Supplementary material for: Targeted next-generation sequencing detects novel gene–phenotype associations and expands the mutational spectrum in cardiomyopathies
Source: PLoS One. 2017 Jul 27;12(7):e0181842. doi: 10.1371/journal.pone.0181842 (PMC5531468; doi:10.1371/journal.pone.0181842)
Supplement: S2 Table — (DOC) [file pone.0181842.s003.doc]

**S2 Table. List of genes included in our panel.**

| **Gene** | **Protein** | **Chromosome** | **Transcripts** | **Number of coding exons included in the panel** | **Associated Phenotypes** |
| --- | --- | --- | --- | --- | --- |
| *ABCC9* | ATP binding cassette subfamily C member 9 | 12 | NM_005691  NM_020297 | 39 | AF, BrS, DCM |
| *ACTA1* | actin, alpha 1, skeletal muscle | 1 | NM_001100 | 6 | DCM, HCM |
| *ACTC1* | actin, alpha, cardiac muscle 1 | 15 | NM_005159 | 6 | DCM, HCM |
| *ACTN2* | actinin alpha 2 | 1 | NM_001103 | 22 | DCM, HCM |
| *AKAP9* | A-kinase anchoring protein 9 | 7 | NM_005751 | 50 | BrS, LQTS, DCM, HCM |
| *ANK2* | ankyrin 2, neuronal | 4 | NM_001148  NM_001127493 | 50 | AF, BrS, CPVT, LQTS, DCM |
| *ANKRD1* | ankyrin repeat domain 1 | 10 | NM_014391 | 9 | DCM, HCM |
| *BAG3* | BCL2 associated athanogene 3 | 10 | NM_004281 | 4 | DCM |
| *CACNA1C* | calcium voltage-gated channel subunit alpha1 C | 12 | NM_000719  NM_199460  NM_001129829  NM_001129833  NM_001129835  NM_001129837  NM_001129838  NM_001129839  NM_001129840  NM_001167623  NM_001167624  NM_001167625 | 52 | BrS, HCM, LQTS, DCM |
| *CACNA2D1* | calcium voltage-gated channel auxiliary subunit alpha2delta 1 | 7 | NM_000722 | 39 | BrS |
| *CACNB2* | calcium voltage-gated channel auxiliary subunit beta 2 | 10 | NM_000724  NM_201570  NM_201571  NM_201572  NM_201590  NM_201593  NM_201596  NM_201597  NM_001167945 | 21 | BrS |
| *CALM1* | calmodulin 1 (phosphorylase kinase, delta) | 14 | NM_006888 | 6 | CPVT, LQTS |
| *CALM2* | calmodulin 2 (phosphorylase kinase, delta) | 2 | NM_001743 | 6 | CPVT, LQTS |
| *CALR3* | calreticulin 3 | 19 | NM_145046 | 9 | DCM, HCM |
| *CASQ2* | calsequestrin 2 | 1 | NM_001232 | 11 | BrS, CPVT, DCM, HCM |
| *CAV3* | caveolin 3 | 3 | NM_033337 | 2 | HCM, LQTS |
| *CHRM2* | cholinergic receptor muscarinic 2 | 7 | NM_001006626  NM_001006630 | 1 | DCM, HCM |
| *COX15* | COX15 cytochrome c oxidase assembly homolog | 10 | NM_004376  NM_078470 | 10 | HCM |
| *CRYAB* | crystallin alpha B | 11 | NM_001885 | 3 | DCM |
| *CSRP3* | cysteine and glycine rich protein 3 | 11 | NM_003476 | 5 | DCM, HCM |
| *CTF1* | cardiotrophin 1 | 16 | NM_001330 | 3 | DCM |
| *CTNNA3* | catenin alpha 3 | 10 | NM_013266 | 19 | ARVC |
| *DES* | Desmin | 2 | NM_001927 | 9 | ARVC, DCM, HCM |
| *DLG1* | discs large homolog 1, scribble cell polarity complex component | 3 | NM_004087  NM_001098424  NM_001204386  NM_001204387  NM_001204388 | 28 | BrS, DCM, HCM |
| *DMD* | dystrophin | X | NM_000109  NM_004006  NM_004009  NM_004010  NM_004011  NM_004012  NM_004014  NM_004015  NM_004016  NM_004019  NM_004020  NM_004021 | 85 | DCM, HCM, ARVC |
| *DOLK* | dolichol kinase | 9 | NM_014908 | 1 | DCM |
| *DSC2* | desmocollin 2 | 18 | NM_004949  NM_024422 | 17 | ARVC, DCM, HCM |
| *DSG2* | desmoglein 2 | 18 | NM_001943 | 15 | ARVC, BrS, DCM, HCM |
| *DSP* | Desmoplakin | 6 | NM_004415 | 24 | ARVC, BrS, DCM, HCM |
| *DTNA* | dystrobrevin alpha | 18 | NM_001390  NM_001391  NM_001392  NM_032978  NM_032979  NM_032981  NM_001128175  NM_001198938  NM_001198939  NM_001198941  NM_001198942  NM_001198945 | 25 | DCM, HCM |
| *EMD* | Emerin | X | NM_000117 | 6 | DCM, HCM |
| *EYA4* | EYA transcriptional coactivator and phosphatase 4 | 6 | NM_004100  NM_172105 | 21 | DCM |
| *FHL1* | four and a half LIM domains 1 | X | NM_001159699  NM_001159700  NM_001159701  NM_001159702  NM_001159703 | 8 | DCM, HCM |
| *FHL2* | four and a half LIM domains 2 | 2 | NM_001450  NM_201555  NM_201557 | 8 | DCM, HCM |
| *FKRP* | fukutin related protein | 19 | NM_024301 | 1 | DCM |
| *FKTN* | fukutin | 9 | NM_006731 | 9 | DCM |
| *FXN* | Frataxin | 9 | NM_000144  NM_181425  NM_001161706 | 6 | DCM, HCM |
| *GATA5* | GATA binding protein 5 | 20 | NM_080473 | 6 | AF |
| *GATAD1* | GATA zinc finger domain containing 1 | 7 | NM_021167 | 5 | DCM |
| *GJA1* | gap junction protein alpha 1 | 6 | NM_000165 | 1 | AF |
| *GJA5* | gap junction protein alpha 5 | 1 | NM_005266 | 1 | AF |
| *GLA* | galactosidase alpha | X | NM_000169 | 7 | DCM, HCM |
| *GPD1L* | glycerol-3-phosphate dehydrogenase 1-like | 3 | NM_015141 | 8 | BrS |
| *HCN4* | hyperpolarization activated cyclic nucleotide gated potassium channel 4 | 15 | NM_005477 | 8 | AF, BrS, HCM |
| *ILK* | integrin linked kinase | 11 | NM_004517 | 12 | DCM, HCM |
| *JPH2* | junctophilin 2 | 20 | NM_020433 | 5 | AF, DCM, HCM |
| *JUP* | junction plakoglobin | 17 | NM_002230 | 13 | ARVC, BrS, DCM, HCM |
| *KCNA5* | potassium voltage-gated channel subfamily A member 5 | 12 | NM_002234 | 1 | AF |
| *KCND3* | potassium voltage-gated channel subfamily D member 3 | 1 | NM_004980 | 7 | AF, BrS |
| *KCNE1* | potassium voltage-gated channel subfamily E regulatory subunit 1 | 21 | NM_000219 | 1 | AF, LQTS |
| *KCNE2* | potassium voltage-gated channel subfamily E regulatory subunit 2 | 21 | NM_172201 | 1 | AF, BrS, HCM, LQTS |
| *KCNE3* | potassium voltage-gated channel subfamily E regulatory subunit 3 | 11 | NM_005472 | 1 | AF, BrS |
| *KCNE5* | potassium voltage-gated channel subfamily E regulatory subunit 5 | X | NM_012282 | 1 | AF, BrS |
| *KCNH2* | potassium voltage-gated channel subfamily H member 2 | 7 | NM_000238  NM_172056  NM_001204798 | 15 | AF, BrS, DCM, HCM, LQTS |
| *KCNJ2* | potassium voltage-gated channel subfamily J member 2 | 17 | NM_000891 | 1 | AF, BrS, CPVT, DCM, LQTS |
| *KCNJ5* | potassium voltage-gated channel subfamily J member 5 | 11 | NM_000890 | 2 | LQTS |
| *KCNJ8* | potassium voltage-gated channel subfamily J member 8 | 12 | NM_004982 | 2 | BrS, HCM |
| *KCNQ1* | potassium voltage-gated channel subfamily Q member 1 | 11 | NM_000218 | 16 | AF, DCM, HCM, LQTS |
| *LAMA4* | laminin subunit alpha 4 | 6 | NM_002290  NM_001105206  NM_001105207  NM_001105209 | 39 | DCM, HCM |
| *LAMP2* | lysosomal associated membrane protein 2 | X | NM_002294  NM_013995  NM_001122606 | 11 | DCM, HCM |
| *LDB3* | LIM domain binding 3 | 10 | NM_007078  NM_001080115  NM_001080116  NM_001171610  NM_001171611 | 16 | ARVC, DCM, HCM |
| *LMNA* | lamin A/C | 1 | NM_005572  NM_170707  NM_170708 | 15 | AF, ARVC, DCM, HCM |
| *MURC* | muscle related coiled-coil protein | 9 | NM_001018116 | 2 | DCM |
| *MYBPC3* | myosin binding protein C, cardiac | 11 | NM_000256 | 34 | DCM, HCM |
| *MYH6* | myosin, heavy chain 6, cardiac muscle, alpha | 14 | NM_002471 | 37 | DCM, HCM |
| *MYH7* | myosin, heavy chain 7, cardiac muscle, alpha | 14 | NM_000257 | 38 | DCM, HCM |
| *MYL2* | myosin light chain 2 | 12 | NM_000432 | 7 | DCM, HCM |
| *MYL3* | myosin light chain 3 | 3 | NM_000258 | 6 | HCM |
| *MYLK2* | myosin light chain kinase 2 | 20 | NM_033118 | 12 | HCM |
| *MYO6* | myosin VI | 6 | NM_004999 | 34 | HCM, DCM |
| *MYOZ2* | myozenin 2 | 4 | NM_016599 | 5 | DCM, HCM |
| *MYPN* | Myopalladin | 10 | NM_032578  NM_001256267 | 21 | DCM, HCM |
| *NDUFV2* | NADH:ubiquinone oxidoreductase core subunit V2 | 18 | NM_021074 | 8 | HCM |
| *NEBL* | nebulette | 10 | NM_006393  NM_213569  NM_001173484 | 32 | DCM, HCM |
| *NEXN* | nexilin F-actin binding protein | 1 | NM_144573 | 12 | DCM, HCM |
| *NOS1AP* | nitric oxide synthase 1 adaptor protein | 1 | NM_014697  NM_001126060 | 10 | LQTS |
| *NPPA* | natriuretic peptide A | 1 | NM_006172 | 3 | AF |
| *NUP155* | nucleoporin 155kDa | 5 | NM_004298  NM_153485 | 35 | AF, DCM |
| *OBSCN* | obscurin, cytoskeletal calmodulin and titin-interacting RhoGEF | 1 | NM_052843  NM_001098623 | 106 | DCM, HCM, ARVC |
| *PDLIM3* | PDZ and LIM domain 3 | 4 | NM_014476  NM_001114107 | 9 | DCM, HCM |
| *PKP2* | plakophilin 2 | 12 | NM_004572 | 14 | ARVC, BrS, DCM, HCM |
| *PLN* | Phospholamban | 6 | NM_002667 | 1 | ARVC, DCM, HCM |
| *PRKAG2* | protein kinase AMP-activated non-catalytic subunit gamma 2 | 7 | NM_016203 | 16 | HCM |
| *PSEN1* | presenilin 1 | 14 | NM_000021 | 10 | DCM |
| *PSEN2* | presenilin 2 | 1 | NM_000447  NM_012486 | 10 | DCM, HCM |
| *RAF1* | Raf-1 proto-oncogene, serine/threonine kinase | 3 | NM002880 | 16 | DCM, HCM |
| *RANGRF* | RAN guanine nucleotide release factor | 17 | NM_016492  NM_001177801  NM_001177802 | 5 | BrS |
| *RBM20* | RNA binding motif protein 20 | 10 | NM_001134363 | 14 | DCM, HCM |
| *RYR2* | ryanodine receptor 2 | 1 | NM_001035 | 105 | AF, ARVC, BrS, CPVT, DCM, HCM, LQTS |
| *SCN5A* | sodium voltage-gated channel alpha subunit 5 | 3 | NM_198056  NM_001099404  NM_001099405  NM_001160160  NM_001160161 | 28 | AF, ARVC, BrS, DCM, HCM, LQTS |
| *SCN1B* | sodium voltage-gated channel beta subunit 1 | 19 | NM_001037  NM_199037 | 5 | AF, BrS, LQTS |
| *SCN2B* | sodium voltage-gated channel beta subunit 2 | 11 | NM_004588 | 4 | AF, BrS |
| *SCN3B* | sodium voltage-gated channel beta subunit 3 | 11 | NM_018400 | 5 | AF, BrS |
| *SCN4B* | sodium voltage-gated channel beta subunit 4 | 11 | NM_174934 | 5 | AF, LQTS |
| *SDHA* | succinate dehydrogenase complex flavoprotein subunit A | 5 | NM_004168 | 14 | DCM |
| *SGCD* | sarcoglycan delta | 5 | NM_000377  NM_172244 | 8 | DCM |
| *SLC25A4* | solute carrier family 25 member 4 | 4 | NM_001151 | 4 | HCM |
| *SLMAP* | sarcolemma associated protein | 3 | NM_007159 | 24 | BrS |
| *SNTA1* | syntrophin alpha 1 | 20 | NM_003098 | 8 | LQTS |
| *SYNE1* | spectrin repeat containing nuclear envelope protein 1 | 6 | NM_033071  NM_182961 | 146 | DCM, HCM, ARVC |
| *TAZ* | Tafazzin | X | NM_000116 | 11 | DCM, HCM |
| *TCAP* | titin-cap | 17 | NM_003673 | 2 | DCM, HCM |
| *TGFB3* | transforming growth factor beta 3 | 14 | NM_003239 | 7 | ARVC, DCM, HCM |
| *TMEM43* | transmembrane protein 43 | 3 | NM_024334 | 12 | ARVC, DCM, HCM |
| *TMPO* | Thymopoietin | 12 | NM_003276  NM_001032283  NM_001032284 | 10 | DCM |
| *TNNC1* | troponin C1, slow skeletal and cardiac type | 3 | NM_003280 | 6 | DCM, HCM, ARVC |
| *TNNI3* | troponin I3, cardiac type | 19 | NM_000363 | 7 | AF, DCM, HCM |
| *TNNT2* | troponin T2, cardiac type | 1 | NM_000364  NM_001001430  NM_001001431  NM_001001432  NM_001276345 | 16 | DCM, HCM |
| *TPM1* | tropomyosin 1 (alpha) | 15 | NM_000366, NM_001018005  NM_001018007  NM_001018020 | 14 | DCM, HCM |
| *TRDN* | Triadin | 6 | NM_006073  NM_001251987 | 42 | CPVT |
| *TRPM4* | transient receptor potential cation channel subfamily M member 4 | 19 | NM_017636 | 24 | BrS, DCM, ARVC |
| *TTN* | Titin | 2 | NM_003319  NM_133378  NM_133379  NM_133432  NM_133437 | 315 | ARVC, DCM, HCM |
| *TTR* | Transthyretin | 18 | NM_000371 | 4 | DCM, HCM |
| *TXNRD2* | thioredoxin reductase 2 | 22 | NM_006440 | 16 | DCM |
| *VCL* | Vinculin | 10 | NM_014000 | 22 | DCM, HCM, ARVC |

The gene names, provided by HGNC, are reported in alphabetical order.

AF = Atrial Fibrillation; ARVC = arrhythmogenic right ventricular cardiomyopathy; BrS = Brugada Syndrome; CPVT = Catecholaminergic Polymorphic Ventricular Tachycardia; DCM = dilated cardiomyopathy; HCM = hypertrophic cardiomyopathy; LQTS = Long QT Syndrome.

The “Associated Phenotypes” column also reported the new associated phenotypes detected in this study (underlined).
