# Supplementary material for: Targeted next-generation sequencing detects novel gene–phenotype associations and expands the mutational spectrum in cardiomyopathies
Source: PLoS One. 2017 Jul 27;12(7):e0181842. doi: 10.1371/journal.pone.0181842 (PMC5531468; doi:10.1371/journal.pone.0181842)
Supplement: S3 Table — (DOC) [file pone.0181842.s004.doc]

**S3 Table. Accession numbers of sequencing data for each patient included in this study submitted to “The European Genome-phenome Archive”.**

| **Patient ID** | **Accession Number** | **Secondary Accession Number** |
| --- | --- | --- |
| 76DCM | EGAN00001553783 | SAMEA104120094 |
| 99DCM | EGAN00001553784 | SAMEA104120095 |
| 310DCM | EGAN00001553785 | SAMEA104120096 |
| 365DCM | EGAN00001553786 | SAMEA104120097 |
| 682DCM | EGAN00001553787 | SAMEA104120098 |
| 737DCM | EGAN00001553788 | SAMEA104120099 |
| 968DCM | EGAN00001553789 | SAMEA104120100 |
| 1060DCM | EGAN00001553790 | SAMEA104120101 |
| 1329DCM | EGAN00001553791 | SAMEA104120102 |
| 1584DCM | EGAN00001553792 | SAMEA104120103 |
| 1669DCM | EGAN00001553793 | SAMEA104120104 |
| 1717DCM | EGAN00001553794 | SAMEA104120105 |
| 1718DCM | EGAN00001553795 | SAMEA104120106 |
| 1801DCM | EGAN00001553796 | SAMEA104120107 |
| 1816DCM | EGAN00001553797 | SAMEA104120108 |
| 1838DCM | EGAN00001553798 | SAMEA104120109 |
| 1173HCM | EGAN00001553799 | SAMEA104120110 |
| 1657HCM | EGAN00001553800 | SAMEA104120111 |
| 1661HCM | EGAN00001553801 | SAMEA104120112 |
| 1674HCM | EGAN00001553802 | SAMEA104120113 |
| 1685HCM | EGAN00001553803 | SAMEA104120114 |
| 1699HCM | EGAN00001553804 | SAMEA104120115 |
| 1721HCM | EGAN00001553805 | SAMEA104120116 |
| 1739HCM | EGAN00001553806 | SAMEA104120117 |
| 1740HCM | EGAN00001553807 | SAMEA104120118 |
| 1741HCM | EGAN00001553808 | SAMEA104120119 |
| 1776HCM | EGAN00001553809 | SAMEA104120120 |
| 1798HCM | EGAN00001553810 | SAMEA104120121 |
| 1832HCM | EGAN00001553811 | SAMEA104120122 |
| 1833HCM | EGAN00001553812 | SAMEA104120123 |
| 1662ARVC | EGAN00001553813 | SAMEA104120124 |
| 1665ARVC | EGAN00001553814 | SAMEA104120125 |
| 1666ARVC | EGAN00001553815 | SAMEA104120126 |
| 1708ARVC | EGAN00001553816 | SAMEA104120127 |
| 1751ARVC | EGAN00001553817 | SAMEA104120128 |
| 1812ARVC | EGAN00001553818 | SAMEA104120129 |
| 1825ARVC | EGAN00001553819 | SAMEA104120130 |
| 1830ARVC | EGAN00001553820 | SAMEA104120131 |
