# Supplementary material for: Targeted next-generation sequencing detects novel gene–phenotype associations and expands the mutational spectrum in cardiomyopathies
Source: PLoS One. 2017 Jul 27;12(7):e0181842. doi: 10.1371/journal.pone.0181842 (PMC5531468; doi:10.1371/journal.pone.0181842)
Supplement: S4 Table — (DOC) [file pone.0181842.s005.doc]

**S4 Table. Number of total variants detected in all patients, according to variant classification.**

| **Patient ID** | **non synonymous** | **nonsense** | **in-frame** | **Frameshift** | **splicing** |
| --- | --- | --- | --- | --- | --- |
| 76DCM | 83 | 2 | 1 | 1 | 0 |
| 99DCM | 89 | 1 | 0 | 1 | 0 |
| 310DCM | 80 | 1 | 2 | 0 | 0 |
| 365DCM | 76 | 0 | 1 | 0 | 0 |
| 682DCM | 99 | 0 | 0 | 0 | 0 |
| 737DCM | 93 | 0 | 1 | 0 | 0 |
| 968DCM | 65 | 0 | 1 | 0 | 0 |
| 1060DCM | 79 | 0 | 1 | 0 | 2 |
| 1329DCM | 81 | 0 | 1 | 0 | 0 |
| 1584DCM | 78 | 0 | 0 | 1 | 0 |
| 1669DCM | 93 | 2 | 1 | 0 | 1 |
| 1717DCM | 108 | 1 | 1 | 0 | 1 |
| 1718DCM | 84 | 0 | 0 | 0 | 0 |
| 1801DCM | 74 | 0 | 1 | 0 | 2 |
| 1816DCM | 77 | 1 | 2 | 0 | 0 |
| 1838DCM | 80 | 0 | 0 | 0 | 1 |
| 1173HCM | 98 | 2 | 1 | 0 | 0 |
| 1657HCM | 75 | 0 | 1 | 0 | 1 |
| 1661HCM | 107 | 0 | 0 | 1 | 1 |
| 1674HCM | 97 | 0 | 1 | 0 | 1 |
| 1685HCM | 73 | 0 | 1 | 0 | 1 |
| 1699HCM | 100 | 1 | 2 | 0 | 1 |
| HCM1721 | 96 | 0 | 1 | 0 | 2 |
| 1739HCM | 87 | 0 | 0 | 0 | 0 |
| 1740HCM | 91 | 0 | 0 | 0 | 1 |
| 1741HCM | 69 | 0 | 0 | 0 | 0 |
| 1776HCM | 95 | 1 | 1 | 0 | 0 |
| 1798HCM | 76 | 1 | 1 | 0 | 1 |
| 1832HCM | 81 | 0 | 1 | 0 | 0 |
| 1833HCM | 81 | 2 | 0 | 0 | 0 |
| 1662ARVC | 87 | 0 | 1 | 0 | 1 |
| 1665ARVC | 79 | 0 | 1 | 0 | 1 |
| 1666ARVC | 95 | 0 | 1 | 1 | 0 |
| 1708ARVC | 100 | 0 | 0 | 0 | 0 |
| 1751ARVC | 70 | 0 | 1 | 0 | 2 |
| 1812ARVC | 96 | 1 | 1 | 0 | 0 |
| 1825ARVC | 94 | 2 | 1 | 1 | 1 |
| 1830ARVC | 100 | 0 | 0 | 0 | 2 |
