# Supplementary material for: Targeted next-generation sequencing detects novel gene–phenotype associations and expands the mutational spectrum in cardiomyopathies
Source: PLoS One. 2017 Jul 27;12(7):e0181842. doi: 10.1371/journal.pone.0181842 (PMC5531468; doi:10.1371/journal.pone.0181842)
Supplement: S5 Table — (DOC) [file pone.0181842.s006.doc]

**S5 Table. Rare variants detected in our patients.**

| ***Gene*** | ***Genomic***  ***Position*** | ***Transcript*** | ***Nucleotide*** | ***Protein*** | ***dbSNP*** | ***MAF*** | ***Cardiac***  ***Phenotype*** |
| --- | --- | --- | --- | --- | --- | --- | --- |
| ***ACTC1*** | **chr15:35084392** | **NM_005159** | **c.707C>T** | **S236F** | **-** | **-** | **HCM** |
| *AKAP9* | chr7: 91643610 | NM_005751 | c.3580G>A | A1194T | rs139965373 | 0.0006 | HCM |
| ***AKAP9*** | **chr7:91726576** | **NM_005751** | **c.10303C>T** | **R3435X** | **-** | **-** | **DCM** |
| *AKAP9* | chr7:91727479 | NM_005751 | c.10664A>T | D3555V | rs139046510 | 0.0009 | DCM |
| ***AKAP9*** | **chr7:91737871** | **NM_005751** | **c.11610C>G** | **Y3870X** | **rs757753258** | **4.06x10^-6^** | **HCM** |
| *AKAP9* | chr7:91739456 | NM_005751 | c.11707G>A | A3903T | rs201298286 | 0.0001 | HCM |
| *ANK2* | chr4:114195737 | NM_001127493 | c.1552C>T | R518W | rs756495481 | 3.97x10^-5^ | DCM |
| *ANK2* | chr4:114267117 | NM_001127493 | c.4283C>T | T1428M | rs142534126 | 0.0001 | DCM |
| *ANK2* | chr4:114286207 | NM_001127493 | c.4619T>A | V1540D | rs66785829 | 0.002 | DCM |
| *ANK2* | chr4:114276330 | NM_001148 | c.6556G>A | E2186K | rs745422554 | 4.07x10^-6^ | HCM |
| *ANK2* | chr4:114278820 | NM_001148 | c.9046G>A | E3016K | rs149963885 | 0.0006 | DCM |
| *BAG3* | chr10:121432002 | NM_004281 | c.743A>G | H248R | rs369947845 | 2.85x10^-5^ | HCM |
| *BAG3* | chr10:121432040 | NM_004281 | c.781C>T | R261W | rs548032105 | 3.26x10^-5^ | HCM |
| *CACNA1C* | chr12:2788668 | NM_000719 | c.5150C>G | A1717G | rs201492706 | 0.0008 | DCM |
| *CACNA1C* | chr12:2789641 | NM_001167625 | c.5524G>A | E1842K | - | - | HCM |
| *CHRM2* | chr7:136700385 | NM_001006626 | c.773A>G | N258S | rs142006633 | 0.0013 | HCM |
| *CSRP3* | chr11:19209713 | NM_003476 | c.251C>T | T84M | rs777327517 | 2.03x10^-5^ | HCM |
| *DES* | chr2:220285224 | NM_001927 | c.743G>A | R248H | rs375906682 | 8.19x10^-6^ | HCM |
| *DLG1* | chr3:196842808 | NM_004087 | c.1532G>A | R511H | rs748649814 | 1.23x10^-5^ | DCM |
| ***DLG1*** | **chr3:196812473** | **NM_004087** | **c.1915G>A** | **G639R** | **rs369412843** | **8.12x10^-6^** | **HCM** |
| *DLG1* | chr3:196792663 | NM_004087 | c.2215G>T | V739L | rs148283553 | 0.0008 | DCM |
| *DMD* | chrX:32632565 | NM_004009 | c.1325A>G | H442R | rs72468699 | 0.0003 | DCM |
| *DMD* | chrX:32613880 | NM_000109 | c.1584A>T | Q528H | - | - | DCM |
| ***DMD*** | **chrX:32456458** | **NM_004009** | **c.3959G>A** | **R1320H** | **rs768990357** | **2.24x10^-5^** | **ARVC** |
| *DMD* | chrX:31986499 | NM_004009 | c.6559C>T | R2187W | rs149322279 | 0.0004 | DCM |
| ***DSP*** | **chr6:7569522** | **NM_004415** | **c.1524dupG** | **V508fs** | **-** | **-** | **DCM** |
| ***DSP*** | **chr6:7580243** | **NM_004415** | **c.3820G>C** | **A1274P** | **-** | **-** | **DCM** |
| *HCN4* | chr15:73615912 | NM_005477 | c.2522C>T | S841L | rs200546024 | 0.0001 | HCM |
| *KCNJ8* | chr12:21926288 | NM_004982 | c.263C>G | A88G | rs117808169 | 0.0003 | HCM |
| *KCNQ1* | chr11:2608850 | NM_000218 | c.1179G>T | K393N | rs12720457 | 0.001 | DCM |
| *LAMA4* | chr6:112469404 | NM_002290 | c.2287G>T | D763Y | rs370369688 | 1.44x10^-5^ | DCM |
| *LAMA4* | chr6:112463419 | NM_002290 | c.2548G>A | A850T | rs144123257 | 7.22x10^-5^ | DCM |
| *LAMA4* | chr6:112443256 | NM_002290 | c.4415G>A | R1472H | rs140346737 | 9.39x10^-5^ | HCM |
| ***LAMP2*** | **chrX:119576454** | **NM_013995** | **c.928G>A** | **V310I** | **rs104894858** | **-** | **HCM** |
| ***LMNA*** | **chr1:156100468** | **NM_170708** | **c.667_687dup** | **L140_A146dup** | **-** | **-** | **DCM** |
| ***LMNA*** | **chr1:156106964** | **NM_170708** | **c.1549C>T** | **Q517X** | **-** | **-** | **DCM** |
| ***MYBPC3*** | **chr11: 47371475** | **NM_000256** | **c.506-2A>C** | **-** | **rs397516057** | **-** | **HCM** |
| ***MYBPC3*** | **chr11:47369407** | **NM_000256** | **c.821+1G>A** | **-** | **rs397516073** | **2.98x10^-5^** | **HCM** |
| ***MYBPC3*** | **chr11:47367758** | **NM_000256** | **c.1090G>A** | **A364T** | **-** | **-** | **HCM** |
| ***MYBPC3*** | **chr11:47360197** | **NM_000256** | **c.2182G>T** | **E728X** | **rs397515954** | **-** | **HCM** |
| *MYBPC3* | chr11:47353695 | NM_000256 | c.3742G>A | G1248R | rs202147520 | 3.25x10^-5^ | DCM |
| *MYH6* | chr14:23859394 | NM_002471 | c.3604G>A | V1202M | rs368451573 | 0.0002 | HCM |
| *MYH6* | chr14:23858099 | NM_002471 | c.4144A>G | I1382V | - | - | DCM |
| *MYH6* | chr14:23854154 | NM_002471 | c.5260G>C | E1754Q | rs372270600 | 8.12x10^-6^ | HCM |
| ***MYH7*** | **chr14:23895233** | **NM_000257** | **c.2102G>A** | **G701D** | **-** | **-** | **HCM** |
| ***MYH7*** | **chr14:23886383** | **NM_000257** | **c.4498C>T** | **R1500W** | **rs45544633** | **-** | **DCM** |
| *MYH7* | chr14:23884476 | NM_000257 | c.5287G>A | A1763T | rs727504355 | 6.85x10^-5^ | HCM |
| ***MYH7*** | **chr14:23884353** | **NM_000257** | **c.5410G>A** | **A1804T** | **rs730880818** | **4.06x10^-6^** | **DCM** |
| *MYL2* | chr12:111350901 | NM_000432 | c.401A>C | E134A | rs143139258 | 0.0002 | HCM |
| *MYO6* | chr6:76608128 | NM_004999 | c.3176G>C | R1059T | rs202214380 | 0.0002 | DCM |
| *NEBL* | chr10:21177128 | NM_006393 | c.267C>G | Y89X | rs147622517 | 0.0013 | DCM |
| *NEBL* | chr10:21106567 | NM_006393 | c.2110C>G | L704V | rs751282061 | 4.07x10^-6^ | HCM |
| ***NEXN*** | **chr1:78401657** | **NM_144573** | **c.1398_1400delAAT** | **I467del** | **-** | **-** | **HCM** |
| *NUP155* | chr5:37309255 | NM_153485 | c.2743A>G | N915D | rs746312378 | 3.97x10^-5^ | DCM |
| ***OBSCN*** | **chr1:228400286** | **NM_052843** | **c.802G>T** | **E268X** | **-** | **-** | **HCM** |
| *OBSCN* | chr1:228401183 | NM_052843 | c.1030G>C | V344L | rs569727649 | 0.0007 | DCM |
| *OBSCN* | chr1:228404169 | NM_052843 | c.2143G>A | E715K | rs763115286 | 0.0007 | HCM |
| *OBSCN* | chr1:228432108 | NM_001098623 | c.3317C>A | T1106K | rs368159429 | 3.65x10^-5^ | DCM |
| *OBSCN* | chr1:228456398 | NM_001098623 | c.5029G>A | V1677M | rs368761334 | 0.0002 | HCM |
| *OBSCN* | chr1:228481940 | NM_001098623 | c.11219C>T | A3740V | rs374797523 | 3.6x10^-5^ | ARVC |
| *OBSCN* | chr1:228495983 | NM_001098623 | c.12638G>A | R4213H | rs56306215 | 0.0013 | HCM |
| *OBSCN* | chr1:228496013 | NM_001098623 | c.12668C>T | T4223M | rs147355183 | 0.0005 | DCM |
| *OBSCN* | chr1: 228505257 | NM_001098623 | c.13654G>A | G4552S | rs201223554 | 0.0006 | DCM |
| ***OBSCN*** | **chr1:228525823** | **NM_001098623** | **c.16979C>T** | **A5660V** | **rs191098985** | **0.0006** | **DCM** |
| *OBSCN* | chr1:228526619 | NM_001098623 | c.17150G>A | S5717N | rs376194851 | 0.0005 | DCM |
| ***OBSCN*** | **chr1:228527758** | **NM_001098623** | **c.17371G>C** | **A5791P** | **rs200362121** | **0.0004** | **ARVC** |
| ***OBSCN*** | **chr1:228557681** | **NM_001098623** | **c.20006G>A** | **R6669H** | **rs373638525** | **9.4x10^-5^** | **DCM** |
| *OBSCN* | chr1:228560426 | NM_001098623 | c.21947A>C | K7316T | rs755659827 | 1.45x10^-5^ | HCM |
| *OBSCN* | chr1:228562438 | NM_001098623 | c.22648G>A | V7550I | rs143872350 | 0.0002 | ARVC |
| *OBSCN* | chr1:228565209 | NM_001098623 | c.23299G>A | E7767K | rs182714476 | 0.0013 | DCM |
| ***PKP2*** | **chr12:33030842** | **NM_004572** | **c.962_972del**  **TCGGCCAGGCG** | **V321GfsX11** | **-** | **-** | **ARVC** |
| *PKP2* | chr12:33030856 | NM_004572 | c.958A>C | T320P | - | - | ARVC |
| *PKP2* | chr12:33030802 | NM_004572 | c.1012A>G | T338A | rs139851304 | 0.002 | DCM |
| ***PKP2*** | **chr12:32974392** | **NM_004572** | **c.2043delT** | **I681fs** | **-** | **-** | **ARVC** |
| *PKP2* | chr12:32974352 | NM_004572 | c.2083C>T | R695C | rs199583774 | 0.0001 | DCM |
| *PSEN2* | chr1:227071472 | NM_012486 | c.208G>A | G70R | rs139972151 | 5.41x10^-5^ | HCM |
| *PSEN2* | chr1:227075813 | NM_012486 | c.520A>G | M174V | rs61757781 | 0.0006 | HCM |
| *RAF1* | chr3:12660102 | NM_002880 | c.119G>A | R40H | rs192632236 | 0.001 | HCM |
| ***RAF1*** | **chr3:12626632** | **NM_002880** | **c.1657A>C** | **N553H** | **rs745876012** | **4.06x10^-6^** | **HCM** |
| *RBM20* | chr10:112579854 | NM_001134363 | c.2575A>G | M859V | - | - | HCM |
| *RYR2* | chr1:237619993 | NM_001035 | c.1570G>A | E524K | - | - | ARVC |
| *RYR2* | chr1:237670107 | NM_001035 | c.2711A>G | Y904C | rs201131315 | 0.0001 | ARVC |
| *RYR2* | chr1:237780626 | NM_001035 | c.5756G>A | R1919Q | rs199893812 | 0.0001 | DCM |
| *RYR2* | chr1:237789101 | NM_001035 | c.6163T>C | S2055P | rs746490469 | 4.08x10^-6^ | ARVC |
| ***RYR2*** | **chr1:237802395** | **NM_001035** | **c.7009G>C** | **G2337R** | **-** | **-** | **ARVC** |
| *RYR2* | chr1:237923081 | NM_001035 | c.11331G>A | M3777I | - | - | DCM |
| *SDHA* | chr5:256470 | NM_004168 | c.1930G>A | V644M | rs3211483 | 2.44x10^-5^ | DCM |
| *SYNE1* | chr6:152652867 | NM_182961 | c.12953C>T | T4318M | rs200825287 | 0.0005 | DCM |
| *SYNE1* | chr6:152647194 | NM_182961 | c.15337G>A | V5113I | rs139170018 | 0.0007 | HCM |
| *SYNE1* | chr6:152461140 | NM_182961 | c.25403G>A | R8468H | rs143049227 | 0.0002 | DCM |
| *SYNE1* | chr6:152454618 | NM_182961 | c.25794A>G | I8598M | rs761146246 | 4.06x10^-6^ | ARVC |
| *TMEM43* | chr3:14183242 | NM_024334 | c.1150C>G | L384V | rs193922706 | 5.41x10^-5^ | DCM |
| *TNNC1* | chr3:52485839 | NM_003280 | c.238A>T | M80L | - | - | DCM |
| *TNNC1* | chr3:52485308 | NM_003280 | c.469A>T | M157L | rs730880230 | 4.06x10^-6^ | ARVC |
| ***TNNT2*** | **chr1:201334425** | **NM_001276345** | **c.305G>A** | **R102Q** | **rs121964856** | **-** | **HCM** |
| *TRPM4* | chr19:49674841 | NM_017636 | c.865G>A | E289K | rs770369257 | 3-26x10-5 | DCM |
| *TRPM4* | chr19:49703651 | NM_017636 | c.2740A>T | K914X | rs140799936 | 0.001 | DCM |
| *TRPM4* | chr19:49714439 | NM_017636 | c.3553G>A | V1185I | rs758731104 | 1.78x10^-5^ | ARVC |
| *TTN* | chr2:179605964 | NM_003319 | c.10907A>G | N3636S | rs199844346 | 0.0001 | HCM |
| *TTN* | chr2:179498247 | NM_003319 | c.15644A>G | D5215G | rs181902304 | 8.69x10^-5^ | DCM |
| *TTN* | chr2:179485937 | NM_003319 | c.18313G>A | D6105N | rs751407435 | 4.09x10^-6^ | HCM |
| *TTN* | chr2:179476243 | NM_003319 | c.23518C>T | R7840C | rs267599049 | 6.14x10^-5^ | HCM |
| *TTN* | chr2:179474201 | NM_003319 | c.24641T>C | I8214T | - | - | HCM |
| *TTN* | chr2:179473074 | NM_003319 | c.25341C>G | N8447K | rs199615557 | 0.0003 | ARVC |
| *TTN* | chr2:179472908 | NM_003319 | c.25507A>G | I8503V | rs377571654 | 0.0001 | DCM |
| *TTN* | chr2:179464373 | NM_003319 | c.29060C>T | P9687L | rs200132226 | 0.0002 | DCM |
| *TTN* | chr2:179462367 | NM_003319 | c.30247A>G | M10083V | rs188185141 | 0.00049 | ARVC |
| *TTN* | chr2:179460415 | NM_003319 | c.30469_30471delGAA | E10157del | - | - | DCM |
| *TTN* | chr2:179446322 | NM_003319 | c.39478G>A | D13160N | rs72646870 | 0.0002 | ARVC |
| *TTN* | chr2:179441947 | NM_003319 | c.41920G>A | D13974N | - | - | DCM |
| *TTN* | chr2:179440898 | NM_003319 | c.42766G>C | D14256H | - | - | HCM |
| *TTN* | chr2:179440757 | NM_003319 | c.42907A>G | I14303V | rs367914610 | 0.0001 | HCM |
| ***TTN*** | **chr2:179434235** | **NM_003319** | **c.49429delG** | **V16477fs** | **-** | **-** | **DCM** |
| *TTN* | chr2:179419792 | NM_003319 | c.61199C>T | S20400F | rs146181116 | 0.003 | DCM |
| *TTN* | chr2:179418418 | NM_003319 | C62119G>A | E20707K | rs200503016 | 0.0002 | DCM |
| *TTN* | chr2:179412772 | NM_003319 | c.66386A>G | Y22129C | rs543223589 | 4.7x10^-5^ | DCM |
| *TTN* | chr2:179412442 | NM_003319 | c.66716T>C | V22239A | - | - | HCM |
| *TTN* | chr2:179410548 | NM_003319 | c.68220C>A | F22740L | rs587780983 | 3.26x10^-5^ | ARVC |
| *TTN* | chr2:179401870 | NM_003319 | c.72771G>T | W24257C | rs775769503 | 4.9x10^-5^ | DCM |
| *TTN* | chr2:179401042 | NM_003319 | c.73237T>G | W24413G | rs372304158 | 0.0002 | HCM |
| *TTN* | chr2:179401027 | NM_003319 | c.73252G>C | E24418Q | rs368321767 | 0.0002 | HCM |
| *TTN* | chr2:179399836 | NM_003319 | c.74311T>A | C24771S | rs766439271 | 0.0001 | HCM |
| *TTN* | chr2:179399316 | NM_003319 | c.74831delT | L24944X | - | - | DCM |
| *TTN* | chr2:179398465 | NM_003319 | c.75682A>G | K25228E | rs72629783 | 0.0002 | HCM |
| *TTN* | chr2:179397150 | NM_003319 | c.76997A>G | Y25666C | rs397517789 | 4.47x10^-5^ | DCM |
| *TTN* | chr2:179598139 | NM_133378 | c.12149G>T | W4050L | - | - | DCM |
| *TTN* | chr2:179595064 | NM_133378 | c.14331A>T | Q4777H | rs752853744 | 4.2x10^-6^ | DCM |
| *TTN* | chr2:179593503 | NM_133378 | c.15418C>A | P5140T | rs72648953 | 0.003 | DCM |
| *TTN* | chr2:179590740 | NM_133378 | c.16577T>C | V5526A | - | - | DCM |
| *TTN* | chr2:179590708 | NM_133378 | c.16609G>A | E5537K | rs72648958 | 0.001 | DCM |
| *TTN* | chr2:179578891 | NM_133378 | c.22762A>G | I7588V | rs72648989 | 0.0008 | DCM |
| *TTN* | chr2:179578012 | NM_133378 | c.23117A>G | Y7706C | rs199557654 | 0.0003 | DCM |
| *TTN* | chr2:179567225 | NM_133378 | c.26657G>A | R8886H | rs373355159 | 0.0002 | HCM |
| *TTN* | chr2:179543195 | NM_133378 | c.30124G>A | E10042K | rs376874956 | 0.0002 | DCM |
| *TTN* | chr2:179517223 | NM_133378 | c.31787C>T | A10596V | rs747459623 | 1.63x10^-5^ | DCM |
| *TTN* | chr2:179516680 | NM_133378 | c.32008G>C | E10670Q | - | - | DCM |
| *TTN* | chr2:179641009 | NM_133379 | c.5582G>A | R1861H | rs140914855 | 8.66x10^-5^ | DCM |
| *TTN* | chr2:179638317 | NM_133379 | c.7466A>G | D2489G | - | - | DCM |
| *TTN* | chr2:179638315 | NM_133379 | c.7468delC | R2490fs | - | - | DCM |
| *TTN* | chr2:179613179 | NM_133379 | c.13948C>T | P4650S | rs149748934 | 0.002 | DCM |
| *TTR* | chr18:29178610 | NM_000371 | c.416C>T | T139M | rs28933981 | 0.001 | DCM |
| *VCL* | chr10:75849841 | NM_014000 | c.1237G>A | A413T | rs146278697 | 0.0003 | ARVC |

Last revised May 2017. MAF: minor allele frequency in gnomAD. Potentially pathogenic rare variants are in bold characters.
