# Supplementary material for: Targeted next-generation sequencing detects novel gene–phenotype associations and expands the mutational spectrum in cardiomyopathies
Source: PLoS One. 2017 Jul 27;12(7):e0181842. doi: 10.1371/journal.pone.0181842 (PMC5531468; doi:10.1371/journal.pone.0181842)
Supplement: S7 Table — (DOC) [file pone.0181842.s008.doc]

**S7 Table. List of the mutated genes and number and type of rare variants detected in ARVC patients.**

| ***GENE*** | **Missense** | **Frameshift ins/del** | **Nonframeshift ins/del** | **Stopgain** | **Splice site** | **All type**  **variants** |
| --- | --- | --- | --- | --- | --- | --- |
| *TTN* | 4 | 0 | 0 | 0 | 0 | **4** |
| *RYR2* | 4 | 0 | 0 | 0 | 0 | **4** |
| *OBSCN* | 3 | 0 | 0 | 0 | 0 | **3** |
| *PKP2* | 1 | 2 | 0 | 0 | 0 | **3** |
| *DMD* | 1 | 0 | 0 | 0 | 0 | **1** |
| *SYNE1* | 1 | 0 | 0 | 0 | 0 | **1** |
| *TNNC1* | 1 | 0 | 0 | 0 | 0 | **1** |
| *TRPM4* | 1 | 0 | 0 | 0 | 0 | **1** |
| *VCL* | 1 | 0 | 0 | 0 | 0 | **1** |
| ***Total*** | **17** | **2** | **0** | **0** | **0** | **19** |
