# Supplementary material for: Targeted next-generation sequencing detects novel gene–phenotype associations and expands the mutational spectrum in cardiomyopathies
Source: PLoS One. 2017 Jul 27;12(7):e0181842. doi: 10.1371/journal.pone.0181842 (PMC5531468; doi:10.1371/journal.pone.0181842)
Supplement: S8 Table — (DOC) [file pone.0181842.s009.doc]

**S8 Table. List of the mutated genes and number and type of rare variants detected in DCM patients.**

| **GENE** | **Missense** | **Frameshift ins/del** | **Nonframeshift ins/del** | **Stopgain** | **Splice site** | **All type**  **variants** |
| --- | --- | --- | --- | --- | --- | --- |
| *TTN* | 22 | 2 | 1 | 1 | 0 | **26** |
| *OBSCN* | 8 | 0 | 0 | 0 | 0 | **8** |
| *ANK2* | 4 | 0 | 0 | 0 | 0 | **4** |
| *DMD* | 3 | 0 | 0 | 0 | 0 | **3** |
| *SYNE1* | 2 | 0 | 0 | 0 | 0 | **2** |
| *AKAP9* | 1 | 0 | 0 | 1 | 0 | **2** |
| *DLG1* | 2 | 0 | 0 | 0 | 0 | **2** |
| *DSP* | 1 | 1 | 0 | 0 | 0 | **2** |
| *LAMA4* | 2 | 0 | 0 | 0 | 0 | **2** |
| *LMNA* | 0 | 0 | 1 | 1 | 0 | **2** |
| *MYH7* | 2 | 0 | 0 | 0 | 0 | **2** |
| *PKP2* | 2 | 0 | 0 | 0 | 0 | **2** |
| *RYR2* | 2 | 0 | 0 | 0 | 0 | **2** |
| *TRPM4* | 1 | 0 | 0 | 1 | 0 | **2** |
| *CACNA1C* | 1 | 0 | 0 | 0 | 0 | **1** |
| *KCNQ1* | 1 | 0 | 0 | 0 | 0 | **1** |
| *MYBPC3* | 1 | 0 | 0 | 0 | 0 | **1** |
| *MYH6* | 1 | 0 | 0 | 0 | 0 | **1** |
| *MYO6* | 1 | 0 | 0 | 0 | 0 | **1** |
| *NEBL* | 0 | 0 | 0 | 1 | 0 | **1** |
| *NUP155* | 1 | 0 | 0 | 0 | 0 | **1** |
| *SDHA* | 1 | 0 | 0 | 0 | 0 | **1** |
| *TMEM43* | 1 | 0 | 0 | 0 | 0 | **1** |
| *TNNC1* | 1 | 0 | 0 | 0 | 0 | **1** |
| *TTR* | 1 | 0 | 0 | 0 | 0 | **1** |
| ***Total*** | **62** | **3** | **2** | **5** | **0** | **72** |
