# Supplementary material for: Targeted next-generation sequencing detects novel gene–phenotype associations and expands the mutational spectrum in cardiomyopathies
Source: PLoS One. 2017 Jul 27;12(7):e0181842. doi: 10.1371/journal.pone.0181842 (PMC5531468; doi:10.1371/journal.pone.0181842)
Supplement: S9 Table — (DOC) [file pone.0181842.s010.doc]

**S9 Table. List of the mutated genes and number and type of rare variants detected in HCM patients.**

| **GENE** | **Missense** | **Frameshift ins/del** | **Nonframeshift ins/del** | **Stopgain** | **Splice site** | **All type**  **variants** |
| --- | --- | --- | --- | --- | --- | --- |
| *TTN* | 12 | 0 | 0 | 0 | 0 | **12** |
| *OBSCN* | 4 | 0 | 0 | 1 | 0 | **5** |
| *MYBPC3* | 1 | 0 | 0 | 1 | 2 | **4** |
| *AKAP9* | 2 | 0 | 0 | 1 | 0 | **3** |
| *BAG3* | 2 | 0 | 0 | 0 | 0 | **2** |
| *MYH6* | 2 | 0 | 0 | 0 | 0 | **2** |
| *MYH7* | 2 | 0 | 0 | 0 | 0 | **2** |
| *PSEN2* | 2 | 0 | 0 | 0 | 0 | **2** |
| *RAF1* | 2 | 0 | 0 | 0 | 0 | **2** |
| *ACTC1* | 1 | 0 | 0 | 0 | 0 | **1** |
| *ANK2* | 1 | 0 | 0 | 0 | 0 | **1** |
| *CACNA1C* | 1 | 0 | 0 | 0 | 0 | **1** |
| *CHRM2* | 1 | 0 | 0 | 0 | 0 | **1** |
| *CSRP3* | 1 | 0 | 0 | 0 | 0 | **1** |
| *DES* | 1 | 0 | 0 | 0 | 0 | **1** |
| *DLG1* | 1 | 0 | 0 | 0 | 0 | **1** |
| *HCN4* | 1 | 0 | 0 | 0 | 0 | **1** |
| *KCNJ8* | 1 | 0 | 0 | 0 | 0 | **1** |
| *LAMA4* | 1 | 0 | 0 | 0 | 0 | **1** |
| *LAMP2* | 1 | 0 | 0 | 0 | 0 | **1** |
| *MYL2* | 1 | 0 | 0 | 0 | 0 | **1** |
| *NEBL* | 1 | 0 | 0 | 0 | 0 | **1** |
| *NEXN* | 0 | 0 | 1 | 0 | 0 | **1** |
| *RBM20* | 1 | 0 | 0 | 0 | 0 | **1** |
| *SYNE1* | 1 | 0 | 0 | 0 | 0 | **1** |
| *TNNT2* | 1 | 0 | 0 | 0 | 0 | **1** |
| ***Total*** | **45** | **0** | **1** | **3** | **2** | **51** |
