# Supplementary material for: Targeted next-generation sequencing detects novel gene–phenotype associations and expands the mutational spectrum in cardiomyopathies
Source: PLoS One. 2017 Jul 27;12(7):e0181842. doi: 10.1371/journal.pone.0181842 (PMC5531468; doi:10.1371/journal.pone.0181842)
Supplement: S11 Table — (DOC) [file pone.0181842.s012.doc]

**S11 Table. Potentially Pathogenic rare variants according to *in silico* prediction tools.**

| **Gene** | | **Variant** | **Grantham’s score** | **SIFT** | **PROVEAN** | **Polyphen-2** | **Mutation**  **Taster** | **Phast Cons** | **PhyloP Score** | **ClinVar** | | **HGMD** | **References*** |
| --- | --- | --- | --- | --- | --- | --- | --- | --- | --- | --- | --- | --- | --- |
| *ACTC1* | S236F | | 155 | DA | DE | PSD | DC | 1 | 5.846 | - | - | |  |
| *AKAP9* | R3435X | | - | - | - | - | DC | 0.118 | 0.719 | - | - | |  |
| *AKAP9* | Y3870X | | - | - | - | - | DC | 0.902 | -0.679 | - | - | |  |
| *DLG1* | G639R | | 125 | DA | DE | PBD | DC | 1 | 5.777 | - | - | |  |
| *DMD* | R1320H | | 29 | DA | DE | PBD | DC | 1 | 4.996 | **-** | **-** | |  |
| *DSP* | V508fs | | - | - | - | - | DC | - | - | - | - | |  |
| *DSP* | A1274P | | 27 | DA | DE | PBD | DC | 0.515 | 4.182 | - | - | |  |
| *LAMP2* | V310I | | 29 | TO | NE | PSD | DC | 1 | 3.503 | P | CM057189 | |  |
| *LMNA* | Q517X | | - | - | - | - | DC | 1 | 5.642 | - | CM1210612 | |  |
| *LMNA* | L140_A146dup | | - | - | **-** | **-** | **-** | **-** | - | - | - | | [52] |
| *MYBPC3* | c.506-2A>C | | - | - | - | - | DC | 1 | 4.745 | P | - | |  |
| *MYBPC3* | c.821+1G>A | | - | - | - | - | DC | 1 | 4.399 | P, LP | CS982276 | |  |
| *MYBPC3* | A364T | | 58 | DA | DE | PSD | DC | 1 | 4.009 | - | - | |  |
| *MYBPC3* | E728X | | - | - | - | - | DC | 1 | 5.623 | P | - | |  |
| *MYH7* | G701D | | 94 | DA | DE | PBD | DC | 1 | 5.899 | - | - | |  |
| *MYH7* | R1500W | | 101 | DA | DE | PBD | DC | 0.998 | 0.219 | P, LP | CM045547 | |  |
| *MYH7* | A1804T | | 58 | DA | DE | PBD | DC | 1 | 6.019 | P | - | |  |
| *NEXN* | I467del | | - | - | - | - | DC | 1 | - | - | - | |  |
| *OBSCN* | E268X | | - | - | - | - | DC | 0.996 | 3.432 | - | - | |  |
| *OBSCN* | A5660V | | 192 | DA | DE | PBD | DC | 1 | 5.545 | - | - | |  |
| *OBSCN* | A5791P | | 27 | DA | DE | PBD | DC | 1 | 5.785 | **-** | **-** | |  |
| *OBSCN* | R6669H | | 29 | DA | DE | PBD | DC | 0.997 | 3.774 | - | - | |  |
| *PKP2* | V321GfsX11 | | - | - | - | - | DC | - | - | - | - | |  |
| *PKP2* | I681fs | | - | - | - | - | DC | 0.91 | -0.485 | - | - | |  |
| *RAF1* | N553H | | 68 | DA | DE | PBD | DC | 1 | 2.431 | - | - | |  |
| *RYR2* | G2337R | | 125 | DA | DE | PBD | DC | 1 | 5.829 | - | - | |  |
| *TNNT2* | R102Q | | 43 | DA | DE | PBD | DC | 1 | 4.206 | P, LP | CM951218 | |  |
| *TTN* | V16477fs | | - | - | - | - | DC | 1 | 3.268 | - | - | |  |

Last revised May 2017. SIFT: Sorting Intolerant From Tolerant; PROVEAN: PROtein Variation Effect ANalyzer; HGMD: Human Gene Mutation Database; DA: Damaging; TO: Tolerated; DE: Deleterious; NE: Neutral; PBD: Probably Damaging; PSD: Possibly Damaging; DC: Disease Causing; B: Benign; LB: Likely Benign; P: Pathogenic; LP: Likely Pathogenic; VUS: Variant of Uncertain Significance; *References: published data supporting the variant pathogenicity.
